# Tracking Protein Misfolding and Oligomerization: A Temperature-Controlled Ion Mobility-Mass Spectrometry Approach

**DOI:** 10.1021/acs.analchem.5c06100

**Published:** 2026-05-13

**Authors:** Despoina Svingou, Luke McAlary, Julian Alexander Harrison, Renato Zenobi

**Affiliations:** † Laboratory of Organic Chemistry, Department of Chemistry and Applied Biosciences, 27219 ETH Zurich, Zurich, 8093 Switzerland; ‡ Molecular Horizons and School of Science, Faculty of Science, Medicine and Health, 8691 University of Wollongong, Wollongong, New South Wales 2522, Australia

## Abstract

Aberrant protein oligomerization is a hallmark of neurodegenerative disorders, yet the conformational and kinetic underpinnings of early aggregation remain poorly understood due to the inability of structural techniques to capture transient, low-abundance oligomeric intermediates. This necessitates the development of a methodology that can characterize the conformational states related to protein unfolding and thus allow for the investigation of the molecular mechanism responsible for disease progression. Here, we demonstrate how temperature-controlled nanoelectrospray ionization (TC-nESI) combined with high-resolution ion mobility–mass spectrometry (IM-MS), surface-induced dissociation (SID), and limited proteolysis can be used to define the misfolding and oligomerization landscape of bovine Cu/Zn superoxide dismutase (SOD1). This integrative approach enables real-time detection of coexisting intermediates, and captures molecular events including metal-induced stability, monomer unfolding and assembly into heterogeneous soluble oligomers. Our results reveal that both holo- and apo-SOD1 undergo dimer dissociation followed by monomer misfolding and assembly into heterogeneous non-native oligomers, and that slow thermal ramping promotes the accumulation of misfolded monomers and higher-order complexes. Apo-SOD1 that lacks stabilizing metal cofactors, forms more compact and kinetically distinct oligomers via monomeric, dimeric and trimeric intermediates. Proteolysis and heat-induced fragmentation identify loops V, VI, VII, and the C-terminus as key labile regions contributing to oligomer interface formation, predominantly through hydrophobic interactions. Our findings establish a mechanistically rich model for early aggregation and demonstrate the capability of TC-nESI-IM-MS to temporally and structurally resolve misfolding transitions and oligomeric populations in a single experiment. This platform provides a framework to dissect oligomerization pathways relevant to neurodegenerative diseases.

## Graphical abstract

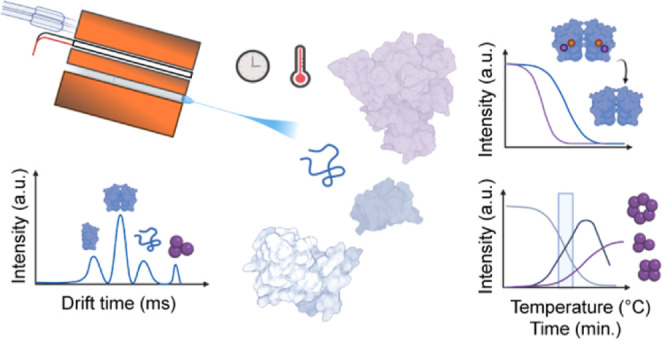

## Introduction

Neurodegenerative diseases encompass a variety of disorders characterized by the progressive disruption of the structure and function of neurons.^1–3^ These debilitating diseases, exhibit a complex mechanism of aberrant protein misfolding, oligomerization, aggregation and ultimately amyloid plaque formation.^2,4^ A major hurdle in elucidating misfolding and early aggregation of proteins has been the lack of techniques to capture and analyze low-abundance intermediates taking part in this dynamic process in real time.^5^ Traditional methods, which mostly yield global information and require long incubation times, often fall short in providing the temporal and structural resolution necessary to study these dynamic and conformationally diverse systems.^5,6^

Oligomeric complexes are commonly studied by spectroscopic methods such as single-molecule fluorescence and nuclear magnetic resonance (NMR) spectroscopy, by size-exclusion chromatography (SEC), or by atomic force microscopy (AFM).^8,9^ However, these low-abundance and transient oligomeric assemblies, often coexist in solution, which presents a significant challenge for traditional biophysical techniques. While powerful, these methodologies typically provide global information rather than unambiguous structural and conformational characterization that would be needed to resolve dynamic oligomeric ensembles.^10^

One technique that has the potential to overcome these challenges is native mass spectrometry (MS). This powerful tool can be used for the characterization of protein complexes and the biophysical properties of their interactions.^11–13^ In native MS, soft nanoelectrospray ionization (nESI) and optimized gas pressures and voltage conditions maintain delicate molecular interactions of even large noncovalent complexes in the gas phase, allowing for detailed structural analysis of heterogeneous protein complex ensembles.^14,15^ Even though structural rearrangements are expected upon desolvation, proteins have been shown to retain many structural aspects upon transfer to the gas phase.^16^ Thus, native MS can serve as a valuable technique that is directly correlated to solution-based methodologies.

Additionally, important information about the conformations adopted by copopulated biomolecular complexes can be derived when native MS is coupled to ion mobility spectrometry (IM-MS), where the overall shape of the ions and their rotationally averaged collision cross section (CCS) can be determined based on their mass, charge and interactions with the drift gas.^17,18^ Especially the development of high-resolution IMS platforms shows great promise in deciphering the conformational landscape of proteins.^19,20^ Another methodology, known as variable-temperature or temperature-controlled nESI (TC-nESI), offers an avenue to probe such processes by accelerating aggregation under defined thermal conditions before ionization.^21,22^ When integrated with high resolution IM-MS, it can provide valuable insights into the thermodynamic properties, stability, folding and interactions of protein complexes.^23–25^ In this study, we explore its potential to induce and access previously unexplored aggregation pathways in real time.

The multitude of pathways a neurodegenerative disease-related protein can take is exemplified here by Cu/Zn superoxide dismutase (SOD1), as it encompasses several molecular characteristics of amyloidogenic proteins, including conformational plasticity, misfolding and metal-regulated stability, folding and assembly (Figure 1).^26–29^ SOD1 is a 32 kDa homodimeric protein that catalyzes the conversion of superoxide, to either H_2_O_2_ or O_2,_ depending on the Cu-oxidation state. Each of its monomers is characterized by a β-barrel scaffold consisting of eight antiparallel β strands and two loops (an electrostatic and a zinc-binding loop). The dimer also contains one copper and one zinc ion per 153-residue subunit.^30,31^ In its fully metalated state, the SOD1 dimer is referred to as holo-SOD1, whereas the demetalated species is known as apo-SOD1. Currently, over 200 SOD1 gene mutations are implicated in amyotrophic lateral sclerosis (ALS).^32,33^ These mutations can affect the structure and stability of the protein, causing dissociation of metal cofactors, the formation of transient neurotoxic oligomers, and finally large insoluble inclusions, all hallmarks of neurodegenerative disease.^34,35^

**Figure 1:**
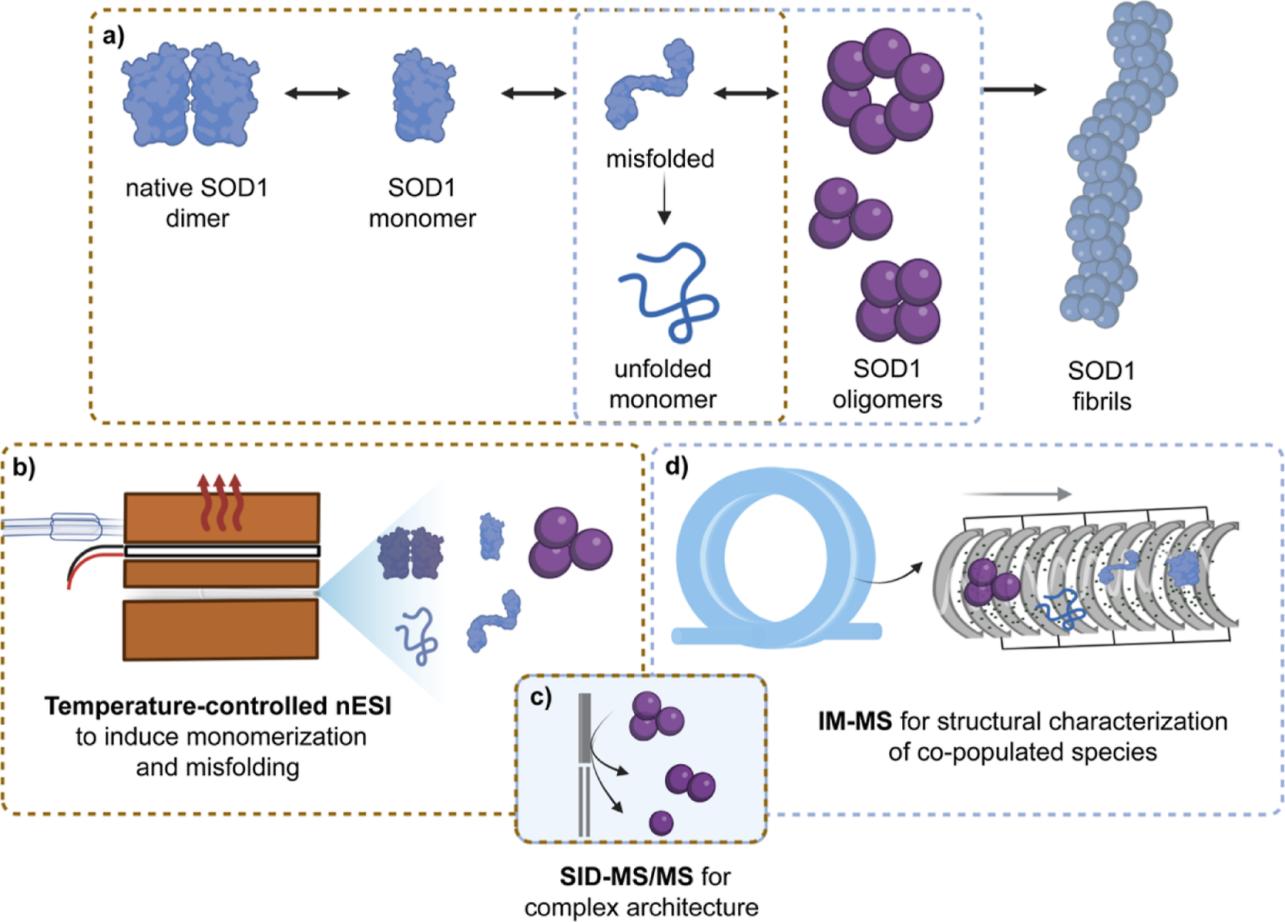
Schematic of methodologies employed to characterize the stages of SOD1 aggregation from native dimer to insoluble fibrils. (a) The SOD1 aggregation pathway proceeds from native dimers to conformationally diverse monomers and subsequently to soluble heterogeneous oligomers, prior to insoluble fibril formation. (b) SOD1 dimer dissociation and monomer misfolding can be induced by controlled thermal ramping using TC-nESI (brown boxes). (c) The architecture of oligomeric complexes can be determined by SID-MS/MS. (d) IM-MS featuring the cyclic IMS design enables structural and conformational characterization of all species (blue boxes).

This work aims to establish a MS-based workflow for probing the early stages of misfolding and oligomerization in neurodegenerative disease–related proteins (Figure 1a). The approach enables precise control of aggregation speed and pathways through temperature-controlled nanoelectrospray ionization (TC-nESI) (Figure 1b). Resulting oligomers are characterized using high-resolution IM-MS, surface-induced dissociation (SID), and limited proteolysis, providing detailed structural and conformational insights at each stage (Figure 1c–d). This generalizable methodological framework allows the investigation of previously uncharacterized intermediates, offering critical understanding of the molecular mechanisms underlying disease progression and aiding in the development of therapeutic strategies.

## Materials and Methods

### Sample Preparation

Protein samples, including jack bean concanavalin A, human serum albumin, alcohol dehydrogenase from *Saccharomyces cerevisiae* and bovine Cu/Zn superoxide dismutase (SOD1) from bovine erythrocytes, were purchased from Merck (Buchs, Switzerland). Buffer exchange and purification of all protein samples was achieved via size exclusion chromatography, on an ÄKTA go system, equipped with a Superdex Increase 200 10/300 column (Cytiva, Massachusetts). All protein samples were sprayed from a 200 mM ammonium acetate (AmAc) solution at pH = 7.4 in concentrations ranging from 20 to 40 μM. These samples were stored at 4 °C until use. Other chemicals used throughout the study include MilliQ water (Millipore, Bedford), ammonium acetate solution, 7.5 M (Merck, Buchs Switzerland) and ethylenediaminetetraacetic acid (EDTA) solution, 0.5 M (Fisher Scientific, Reinach, Switzerland).

Bovine SOD1 samples were subjected to demetalation to obtain the apo species, by incubation in 10 mM EDTA, 50 mM AmAc solution at pH = 3.5 for two days, and subsequent buffer exchange to 200 mM AmAc, pH = 7.4 via SEC, as previously described.^36^

### Native IM-MS Experiments

All native IM-MS experiments were carried out on a SELECT SERIES Cyclic IMS (Waters, Wilmslow, U.K.), equipped with a quadrupole filter that allows isolation of species up to 32000 *m*/*z*, followed by a three-lens SID cell placed in front of the trap collision cell.^37,38^ All samples were sprayed in positive mode from borosilicate glass capillaries (inner diameter of ∼1 μm) that were pulled in-house employing a micropipette puller (P-1000, Sutter Instruments) and fitted with a platinum wire. The mass range was set from 50 to 32000 *m*/*z* for the initial holo-SOD1 study to accommodate higher-mass oligomers and was modified to 50–16000 *m*/*z* for the rest of the experiments. For the heating experiments with a ramp of 1.0 °C min^–1^, the scan rate (i.e., the time over which each spectrum was collected) was set to 1s. When a heating rate of 0.3 °C min^–1^ was employed the scan rate was changed to 2s, to reduce data size during longer acquisitions. For all experiments the mass spectrometer was operating in “V-mode”, while all data were acquired in “Mobility mode”. Primarily, crucial cIMS parameters for high-mass ion transmission and optimal ion mobility separation were considered, as described by Harrison et al.^12^ Operating parameters for all experiments: native IM-MS and collision-induced unfolding tandem MS (CIU-MS/MS), are described in the Supporting Information (Tables S1–S4).

All thermal stability studies were conducted employing a TC-nESI source, built in-house and described in detail by Marchand et al.^22^ In short, temperature regulation of a nESI emitter, placed in a copper block assembly, is achieved by means of a Peltier element and a water-cooling system. Thus, uniform heating of the analyte solution in the nESI emitter is achieved. During the course of the thermal denaturation experiments, the protein solution can be heated from 5- 95 °C at a selected rate. Thermal equilibrium of the system under investigation was ensured by employing slow heating rates between 0.3 and 1.0 °C min^–1^.

CCS calculations for all species were attained by constructing calibration curves, utilizing standards of similar mass, shape and charge state distributions for the probed range (Figures S1 and S12), as previously described.^17,39,41^ More specifically, β- and γ-subunits of paradoxin (extracted from snake venom), concanavalin A, human serum albumin and alcohol dehydrogenase, with concentrations of 10–20 μM in 200 mM AmAc, were chosen as standards.^42,43^

Surface-induced activation experiments were performed by utilizing the three electrode SID assembly (‘Gen 3′ split-lens design comprising of the deflector and extractor/surface electrodes) on mass-selected individual charge states that showed no overlap between oligomeric states.^38^ SID activation energy was ramped in the range of 20–110V in 10–20V steps. At each step, IMS-MS data were collected for 7 min and subsequently combined. Each population was extracted from the corresponding IMS heatmap and deconvoluted to retrieve IMS profiles and dissociation pathways for each oligomeric state.

### Data Acquisition and Processing

All IM-MS data were acquired using MassLynx 4.2 (Waters). Extraction of retention time and drift time profiles, as well as smoothing of acquired spectra, were conducted using Driftscope v3.0 software (Waters) and MassLynx 4.2 (Waters), respectively. Deconvolution of SID-MS/MS data was achieved using UniDec software.^44^ Further data analysis and visualization were performed using Prism 10.1.1 (GraphPad Software) and Adobe Illustrator (26.0.3, Adobe Inc., California). Graphics were created using Biorender.

All thermal denaturation results represent summed data across the entire temperature ramp, unless stated otherwise. Additionally, all melting and formation temperatures reported are defined as the midpoints of sigmoidal or reverse sigmoidal abundance curves for each species, respectively.

## Results and Discussion

The study of aggregation mechanisms responsible for neurodegeneration often involves highly dynamic and transient structures.^45^ This leads to the low abundance of transient oligomers, which are potentially ideal candidates for drug development, but are woefully uncharacterized.^46,47^ Additionally, the underlying kinetics of these multifaceted and dynamic processes are quite challenging to elucidate.^48,49^ Here, we detail how an MS-based assay, which utilizes TC-nESI and IM-MS, can be used to elucidate these transient early aggregation states.

### Method Development and Optimization

To showcase our approach, bovine SOD1 was chosen as a model system due to its high similarity to human SOD1 (82% sequence similarity) and its well-characterized folding behavior, making it an ideal platform to benchmark early aggregation studies.^50,51^ Here, thermal denaturation MS experiments along with high-resolution ion mobility separation, were employed for the simultaneous characterization of thermal stability and oligomer growth mechanisms. However, several methodological adaptations need to be made to study this system with temperature-controlled mass spectrometry. Thus far, most studies using this technique employ heating rates ranging from 1.0 or 2.0 °C min^–1^ to much faster thermal ramping of laser-based methods, to probe solution-phase stability and thermodynamics.^12,23,52,54^ However, the typically used heating rates may not be suitable for studying proteins involved in neurodegenerative disease due to the intricate kinetics of intermediate formation in such systems.^55^ Therefore, we explored different heating rates for a range of temperatures, focusing on their effect on the interplay of SOD1 native dimer dissociation, unfolding and oligomer formation.

Initially, holo-SOD1 was subjected to heating at a rate of 1.0 °C min^–1^ in the temperature range of 25–87 °C (Figure 2). At the beginning of the ramp, the main ions in the spectrum corresponded to the native dimer, with charge states ranging from 9+ to 12+, with 11+ being the most prominent (Figure S2). Interestingly, for the main charge state of the native dimer (2^11+^), three distinct conformations were observed, with CCS of 2826 (native), 3334 and 3666 Å^2^ consistent with partial unfolding of the dimeric structure (Figure S3). The latter two conformers are of low abundance and cannot be identified in the 10+ and 12+ charge states due to overlap with the 5+ and 6+ monomers, respectively (Figure 2).^27^ Other species observed, in lower abundances, include monomer (in charge states ranging from 5+ to 8+) and tetramer (ranging from 14+ to 17+). Only one conformational population was initially observed for both (Figure S2).

**Figure 2:**
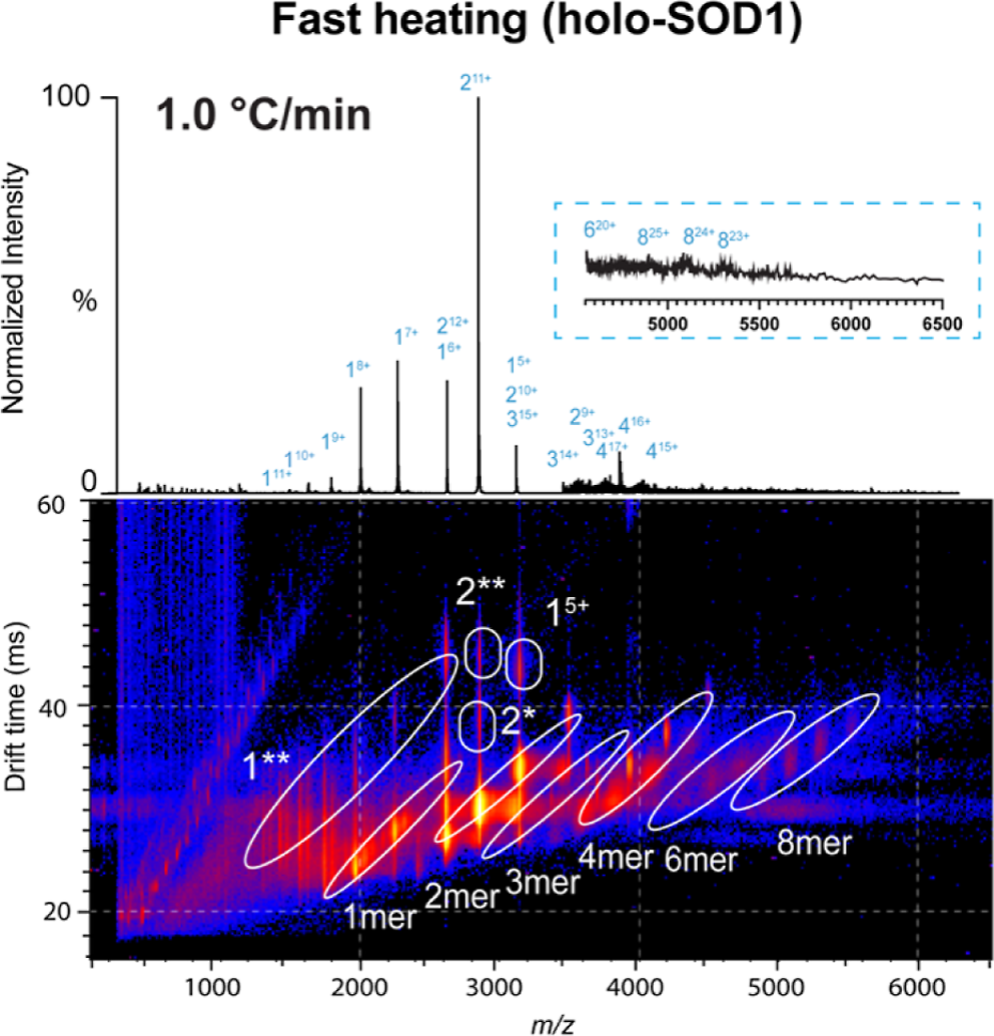
Fast thermal denaturation of holo-SOD1 via TC-nESI-IM-MS. Mass spectrum of holo-SOD1 after fast heating (data summed over the range of 75–85 °C). The data were acquired with a rate of 1.0 °C min^–1^ over the full 25–85 °C ramp. The zoomed-in spectrum shows the *m*/*z* region from 4500 to 6500. The lower *m*/*z* range (50–2300) is x10 magnified, while the higher *m*/*z* range (4000–6500) is ×200 enhanced. The corresponding heat map shows three-dimensional data of the fast ramp offering intensity, drift time and *m*/*z* of all species. The spectra from both the fast and the slow ramp (Figure 3a) contain the same number of scans.

For the fast-heating experiment, at 75 °C, holo-SOD1 first dissociates from dimers into monomers (Figure 2). Throughout this work, misfolded intermediates are denoted as (*), and fully unfolded species as (**), as determined by their charge state and experimentally derived CCS. The IM-MS profile indicated that the monomers formed two populations (Figure 2). Based on CCS and charge state, these species corresponded to folded (1) and unfolded (1**) monomers (Figure S4). The simultaneous appearance of two monomeric populations could stem from localized monomer unfolding prior to dimer dissociation, a phenomenon unlikely in solution and previously attributed to gas-phase dynamics.^56^ However, as the abundance of those populations increased by heating, they can be considered dimer dissociation products resulting from increased internal energy within the complex (Figures 2 and S5). This can yield monomers of variable degrees of unfolding, with the prospect of partial or complete metal loss. Concerning higher-order oligomeric structures, mainly tetramers were observed, along with some very low-abundance trimers, hexamers and octamers (Figure 2). The preliminary data for the typical ramp prompted two prescient questions: can the formation of oligomers be better captured using different heating parameters? How is the monomer folding population related to oligomer assembly? To explore how the kinetics of monomer formation and misfolding influence SOD1 oligomer assembly, we modified the heating rate of the temperature ramp for this aggregation assay.

We used a slightly lower heating rate of 0.3 °C min^–1^ for these TC-nESI experiments in the range of 35–85 °C (Figure 3). At the beginning of the ramp, the main species match those observed in the fast ramp. However, the slightly slower ramp gave rise to a greater amount of misfolded monomer and oligomer formation (Figure 3a). This finding aligns with previous research, suggesting that dimer dissociation and partial unfolding of monomers is key for oligomer formation.^57^ Notably, slower heating shows increased abundance of oligomers containing an odd number of subunits, possibly due to the increased abundance of monomers available for complexation (Figure 3a). To ensure the reproducibility of our results, we performed triplicates of those exploratory thermal ramping experiments on holo-SOD1 (Figures S6 and S7). Overall, this data shows that it is possible to control the speed of monomer and oligomer formation by adjusting the heating rate, revealing previously unseen molecular details for this protein.

**Figure 3:**
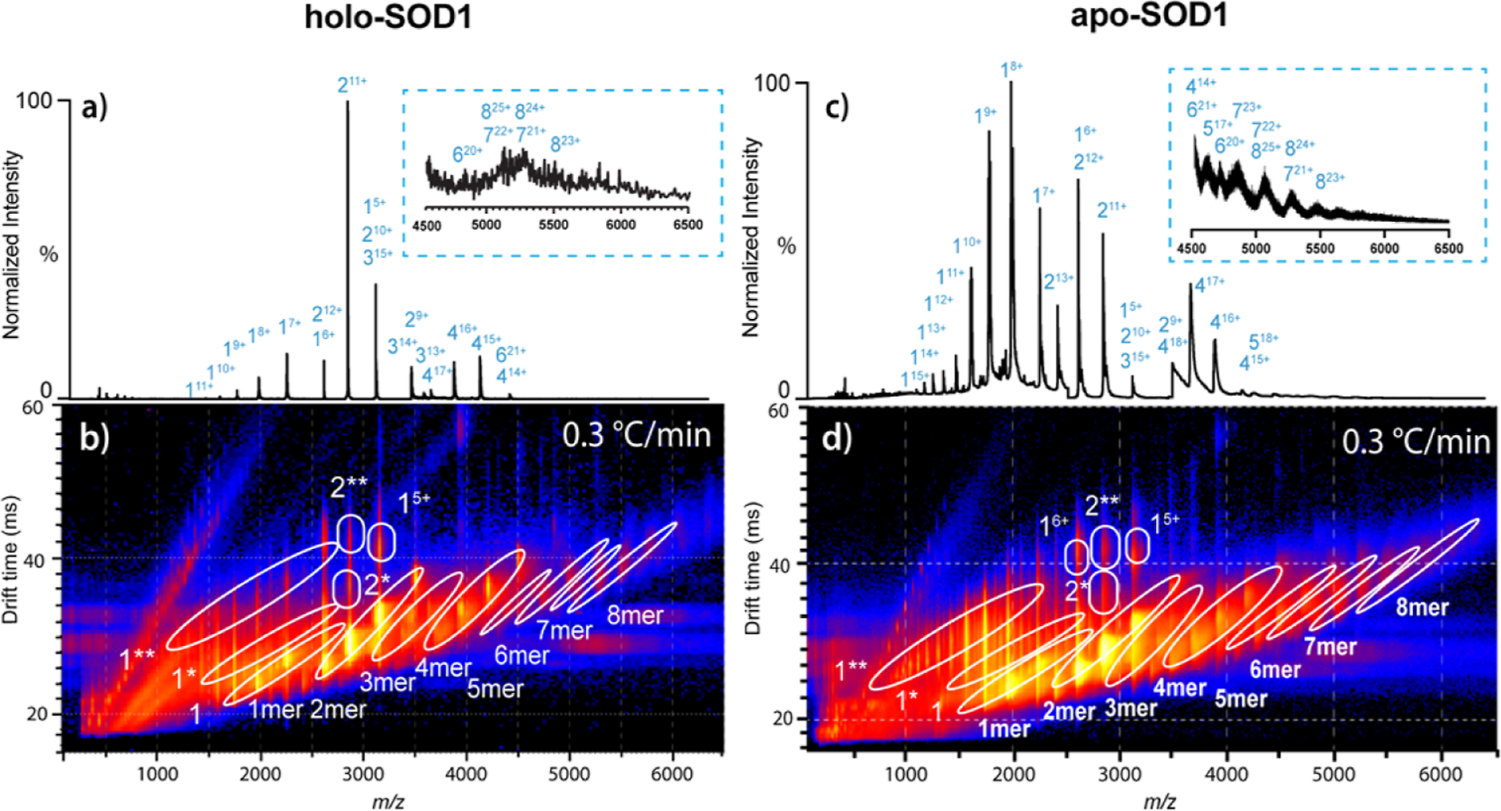
Comparison of holo- and apo-SOD1 during slow thermal denaturation experiments. (a) Mass spectrum of holo-SOD1 after slow heating (summed over the temperature range of 75–85 °C). The data were acquired with a rate of 0.3 °C min^–1^ over the full 35–85 °C ramp. The zoomed-in spectrum shows the *m*/*z* region from 4500 to 6500. The lower *m*/*z* range (50–2300) is 10× magnified, while the higher *m*/*z* range (4500–6500) is ×200 enhanced. Charge states of distinct oligomers are symbolized as O^*n*+^, where O is the number of the subunits and *n* is the charge. The spectra from both the fast (Figure 2) and the slow ramp contain the same number of scans. (b) The corresponding IM-MS heat map showing three-dimensional data of the slow ramp offering intensity, drift time and *m*/*z* of holo-species. (c) Mass spectrum of apo-SOD1 during the thermal denaturation experiment (30–70 °C). Charge states of distinct oligomers are symbolized as O^*n*+^, where O is the number of the subunits and *n* is the charge. (d) All apo-species are shown and labeled on the three-dimensional IM-MS heat map showing intensities, drift times and *m*/*z*.

Because longer heating times resulted in increased monomer and oligomer production, these results provide valuable insights into the kinetics of this process. We investigated this further by performing an isothermal experiment to verify solely the influence of time once dimer dissociation is induced. For this experiment, we utilized the insights provided by the slow temperature ramp and directly exposed the solution to 82 °C to trigger monomer formation. We then followed the profiles of folded and unfolded monomers and holo-oligomers as depicted in Figure S8. The results suggest that dissociation to mainly folded and shortly after to unfolded monomers is achieved after 40 min, deeming it a slow process. Incorporation of mainly tetramers and to some extent dimers into higher oligomeric structures is rather fast (noticeable in the first 10 min), suggesting that holo-oligomer assembly does not necessitate the inclusion of monomers and is faster than the dissociation step for holo-SOD1.

### Monomer Formation from Holo-and Apo-SOD1

To better understand the relationship between SOD1 monomer and oligomer formation, we first tracked the conformational populations of all holo-dimeric and monomeric species during a slow thermal denaturation experiment (35–85 °C, 0.3 °C min^–1^), with corresponding abundance profiles shown in Figure 4a. Upon dimer dissociation, three major monomeric conformational populations emerged; the native monomers (1), a folded (1-M) and a misfolded single-metal intermediate (1*-M) and an unfolded, single-metal species (1**-M). These were accompanied by a population of unfolded apo-monomers (1**apo) with CCS increasing linearly with charge, from 2170 to 3175 Å^2^ (Figures 3a and S10, S11).

**Figure 4:**
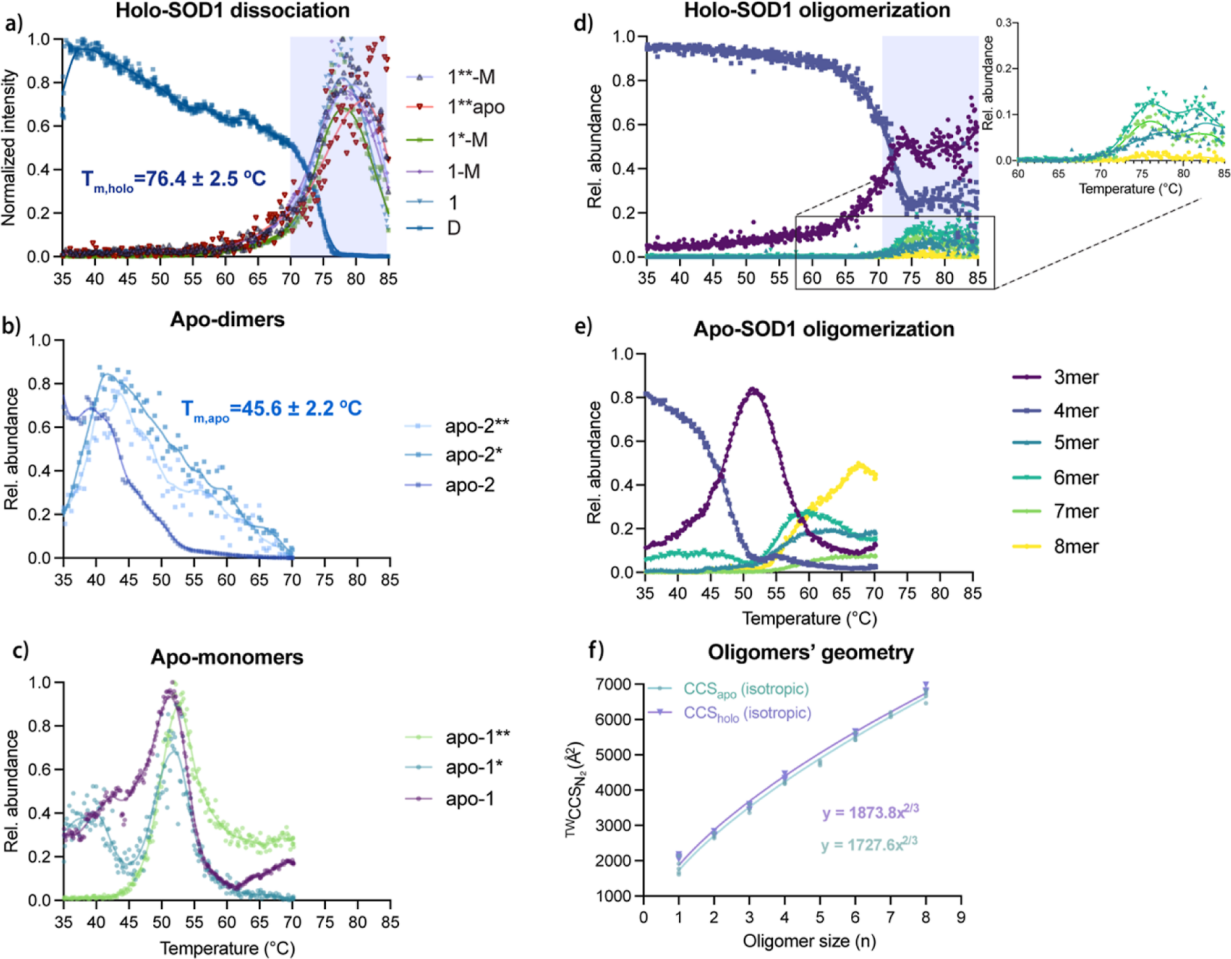
Comparison of thermal denaturation results for holo- and apo-SOD1. (a) Relative abundance profiles as a function of temperature for holo-SOD1 dimers and monomeric populations generated from a slow heating experiment within the range of 35–85 °C and a heating rate of 0.3 °C min^–1^. (b,c) Relative abundance profiles as a function of temperature for apo-SOD1 dimeric and monomeric populations generated from a slow heating experiment within the range of 30–70 °C and a heating rate of 0.3 °C min^–1^ (d,e) Relative abundance of holo- and apo-oligomers across temperatures. The signals of the different oligomeric species are normalized to the sum of all oligomer ions. All signals represent weighted averaged intensities accounting for the contributions of each charge state to their respective population. (f) CCS values of holo- (purple) and apo- (green) oligomers. Experimental CCS (CCSexp) reveal isotropic growth for both states, following the relationship: σ_1_·n^2/3^ (σ_1_ is the CCS of the monomer and n is the number of subunits).

With the conformational landscape established, we followed the thermally driven transitions from dimer to monomer (Figure 4a). All dimeric populations shared a similar abundance profile, decreasing steadily until rapid depletion at 76.4 ± 2.5 °C (*T*_m, holo_), confirming high thermal stability.^58^ Dissociation gave rise to native monomers at 74.0 °C, followed closely by misfolded and unfolded monomers at 75.3 and 76.9 °C, respectively. It is worth mentioning that the main two extended populations are comprised of single-metal monomers, as does a fraction of the native monomer population, with a formation temperature of 76.1 °C. Eventually, the rise of 1** apo-monomers at 84.6 °C is also observed, in lower abundance. Interestingly, all three monomer populations peaked around 80 °C, before collectively decreasing within a narrow range of 83.5–85.0 °C. Unfolded single-metal and apo-monomers persisted till the higher end of this range. This melting event verifies that these species retain some structure and can be considered intermediates, available for higher-order assembly.^10,59^

An important factor affecting SOD1’s misfolding and aggregation propensity is its metalation state. It has been established that mainly the loss of Zn­(II) or both Cu­(II) and Zn­(II) can compromise SOD1’s structural integrity and encourage monomer formation and ultimately aggregation.^60,61^ Hence, we performed offline demetalation by chelation to assess and compare apo-SOD1’s thermal stability and oligomeric profiles with those of holo-SOD1.

In apo-SOD1, without metal cofactors, thermal stability is significantly reduced, as indicated by a melting temperature of 45.6 ± 2.2 °C (*T*_m, apo_) (Figure 4b). The native apo-dimer primarily adopts charge states of 10+ to 12+ (2640–2777 Å^2^), appearing more compact than its holo-analogue due to the loss of metal ions (Figures 3c and S13). However, for the main charge state (11+), two additional conformations, apo-2* and apo-2**, are observed with formation temperatures of 37.7 and 38.1 °C, respectively (Figure 4b). These extended dimer populations plateau at 43 °C, in contrast to the gradual depletion of the native conformer. Interestingly, they account for up to 0.1% of the total apo-dimeric fraction (as calculated by relative intensities), a higher ratio compared with holo-dimers (0.06%) and have a temperature-dependent unfolding pathway in solution. Regarding monomeric species, three monomeric conformations emerge: apo-1 (1668–2168 Å^2^), apo-1* (2049–2897 Å^2^), and apo-1** (2201–3223 Å^2^) (Figure S14). The most compact monomers form at 44.8 °C, while the more extended conformations appear at 49.7 °C, with their intensities peaking at 51 and 52 °C for apo-1* and apo-1**, respectively (Figure 4c). Interestingly, at these temperatures, misfolded apo-1* is the most abundant monomeric species (Figure S15). Its gradual decline coincides with the emergence of higher-order oligomers, suggesting its role as an oligomerization intermediate.

A direct comparison of the two metalation states reveals that the presence of cofactors governs the sequence and temperature dependence of monomer formation. In holo-SOD1, metal binding stabilizes the native dimer, delays dissociation until high temperatures and yields monomeric intermediates that form within a confined temperature range. In contrast, apo-SOD1 lacks this stabilization and therefore undergoes earlier and more gradual monomer formation, producing extended conformers over a broader temperature range. The reduced structural integrity in the apo-state facilitates progressive unfolding, allowing misfolded monomers to accumulate and persist long enough to nucleate higher-order oligomers (Figures 3d and 4c,e). Thus, metal depletion shifts the mechanism from a late unfolding event (holo) to a stepwise, destabilized monomer formation process (apo) that can more readily seed oligomerization.

This also begs the question: can altering the heating time reveal different monomeric populations and potentially different assembly pathways for the more abundant and conformationally variable apo-monomer? Differences in these pathways have proven particularly important when correlating the oligomerization pattern of ALS- associated SOD1 variants with higher aggregation and pathological markers.^10^ To determine how the monomer’s conformations are affected by different heating rates, CIU-MS/MS experiments on monomers were conducted after fast (1.0 °C min^–1^) and slow (0.3 °C min^–1^) heating, respectively (Figure S9). The investigation was carried out by heating the solution in the range of 55–60 °C, where dissociation is expected, prior to mass isolation and stepwise increase of the collision energy in the trap region, while remaining at 60 °C. The 8+ apo-monomer was chosen as the precursor as it represents the most abundant native charge state and does not overlap with any dimeric species. In the short window of an activation potential of 10–40 V, only a slight difference in the gas-phase stability of the two native 8+ monomers was observed. The monomer produced with fast heating shifted to an extended conformation at 22.1 V whereas the slow ramp produced a less stable monomer which underwent the same transition at 19.9 V. This small difference in gas-phase stability is barely sufficient to distinguish these transition states, which is not feasible solely by comparing their respective arrival time distributions (ATDs), in a manner comparable to subtly different antibody glycoforms.^63^

### Oligomer Formation from Holo- and Apo-SOD1

For holo-SOD1, upon monomer formation, a range of oligomeric species were identified, including trimers, tetramers, pentamers, hexamers and octamers (Figure 3a and Table S5). Notably, a rather high concentration of 40 μM (of dimeric SOD1) was chosen, since oligomeric complex production is concentration dependent. However, further increase in concentration was avoided to prevent nonspecific oligomerization. Trimers, hexamers and octamers mirrored the monomers’ abundance pattern, whereas tetramers appeared to follow the dimeric profile (Figure 4d). Intriguingly, almost all observed oligomers (besides trimers) retained metal cofactors, implying that a subset of holo-dimers and tetramers may be directly incorporated into larger complexes.

The remaining monomers were available for the formation of trimers and pentamers. The most abundant oligomeric species seem to be tetramers and trimers, with trimers originating from tetramer dissociation, as confirmed by SID-MS/MS experiments of the 17+ charge state of holo-tetramers prior to heating, because tetramers are most abundant at that stage (Figures 4d and S16). Although this is a gas-phase dissociation of a highly charged tetramer, the symmetric charge partitioning observed upon SID into trimer and monomer is indicative of native-like subunit architecture.^64–66^ This indicates that the tetramer is composed of a trimer and a monomer, rather than a nonspecific dimer of dimers (as they are most abundant in solution), consistent with the dissociation pattern observed during thermal denaturation in solution (Figures S16 and 4d). Intriguingly, trimers were the only oligomers exhibiting metal loss. The pentamer, hexamer, heptamer and octamer all follow a similar pattern with tightly clustered formation temperatures of 73.8, 73.5, 73.7 and 73.8 °C, respectively. Interestingly, their abundance only amounts approximately to 25% of the total oligomer abundance (from 4mers up to 8mers), with hexamers and heptamers slightly more pronounced compared to the pentamers and octamers (Figure 4d).

For apo-SOD1, following monomer formation, oligomer formation becomes apparent, starting with trimers (Figure 4e). The abundance pattern of trimers closely follows that of monomers, indicating rapid assembly once monomers become available. Notably, the trimers are consumed before the appearance of higher structures, with a melting temperature of 56.5 °C. Thus, apo-trimers can be considered intermediates, later incorporated into higher oligomers. Tetramers follow closely the dimer’s pathway. Higher-order structures observed include hexamers, forming first at 55.4 °C. Pentamers appear shortly after with a formation temperature of 56.5 °C. Heptamers, form at 59.8 °C and finally, octamers, form at 60.1 °C. (All charge state distributions and CCS values of the apo-oligomers are listed in Table S6).

In terms of relative abundance, tetramers and trimers are dominating before their gradual decrease and emergence of higher structures, out of which octamers seem to be the most abundant, followed by hexamers, pentamers and finally heptamers. Interestingly, apo-oligomers containing an even number of subunits are favored. This is justified by the higher abundance of dimeric and tetrameric complexes in solution, compared with the monomeric and trimeric intermediates. In this context, it is worth noting that a reverse trend in the formation of hexamers and octamers between the holo- and apo- SOD1 forms suggests that destabilized apo-dimers and monomers have a higher capacity to form larger, more stable oligomers, which aligns with findings showing increased oligomerization in the apo-state of the structurally very similar human SOD1.^59,67^ In terms of structure, geometry, and size, all oligomers exhibit isotropic growth, while in apo-assemblies, more compaction is observed (Figure 4f and Tables S5, S6).

Considering the time needed for the different transitions of apo-SOD1, with a slow heating rate, we observe dimer unfolding and monomer misfolding with a 10-fold higher monomer formation efficiency (calculated as the ratio of maximum intensities of all monomeric to all dimeric species), compared to the holo protein. This can be attributed to the removal of metal ions, thus suppressing their allosteric stabilizing effect on the dimer interface.^69^ This event, in turn, fuels the assembly process of oligomers, a stage that is rather quick for the holo protein, once its very high oligomerization temperature is reached (Figure 4d). Notably, holo-oligomers seem to be (based on their abundances) preferentially organized in complexes containing an even number of subunits, an event concurrent with dimer decrease. However, for apo-oligomers, trimers are identified as additional intermediates of a more gradual oligomerization process occurring at a much lower temperature range of 50–60 °C, whose kinetics are most probably affected by the difference in their assembly mechanism(s) (Figure 4e).

### Investigating the Assembly Mechanism of Apo-Oligomers

Investigating the architecture of transient and low-abundance heterogeneous oligomeric species at the brink of large aggregate formation is inherently challenging. The difficulty increases especially when trying to probe large dynamic structures in the gas-phase, while maintaining solution-phase structural fidelity. To address this, we employed a multifaceted approach combining surface-induced dissociation tandem mass spectrometry (SID-MS/MS) and limited proteolysis at elevated temperatures. This strategy allows access to both solution-phase and gas-phase interactions through proteolytic cleavage, heat-induced fragmentation, and stepwise complex dissociation, respectively.^70^ In doing so, we gain valuable insights into oligomer formation mechanisms and identify potential labile regions that may serve as oligomerization interfaces.

First, SID-MS/MS experiments were performed after a slow temperature ramp (55–60 °C, 0.3 °C min^–1^), ensuring oligomer formation. A wide range of surface collision energies from 20 to 110 V was explored for all oligomeric assemblies, and relative intensities of precursor and dissociation products along this range of potential were traced. The results of the dissociation pathways of each oligomer are depicted in Figure 5, where the cartoons illustrate the most probable dissociation pathway. In such an investigation, quadrupole selection of specific charge states is challenging, as there is significant overlap from different oligomers within the same *m*/*z* window. As charge state selection can dictate charge partitioning and fragmentation efficiency in SID, lower charge states are expected to provide more information about the architecture, retaining the structures of bigger subcomplexes.^65,71^ Hence, for each complex, the lowest of the nonoverlapping charge states was chosen and subjected to SID. The exception was the tetramers, where the 17+ charge state was chosen because lower charge states showed peak overlap with other subunit configurations in the spectrum.

**Figure 5:**
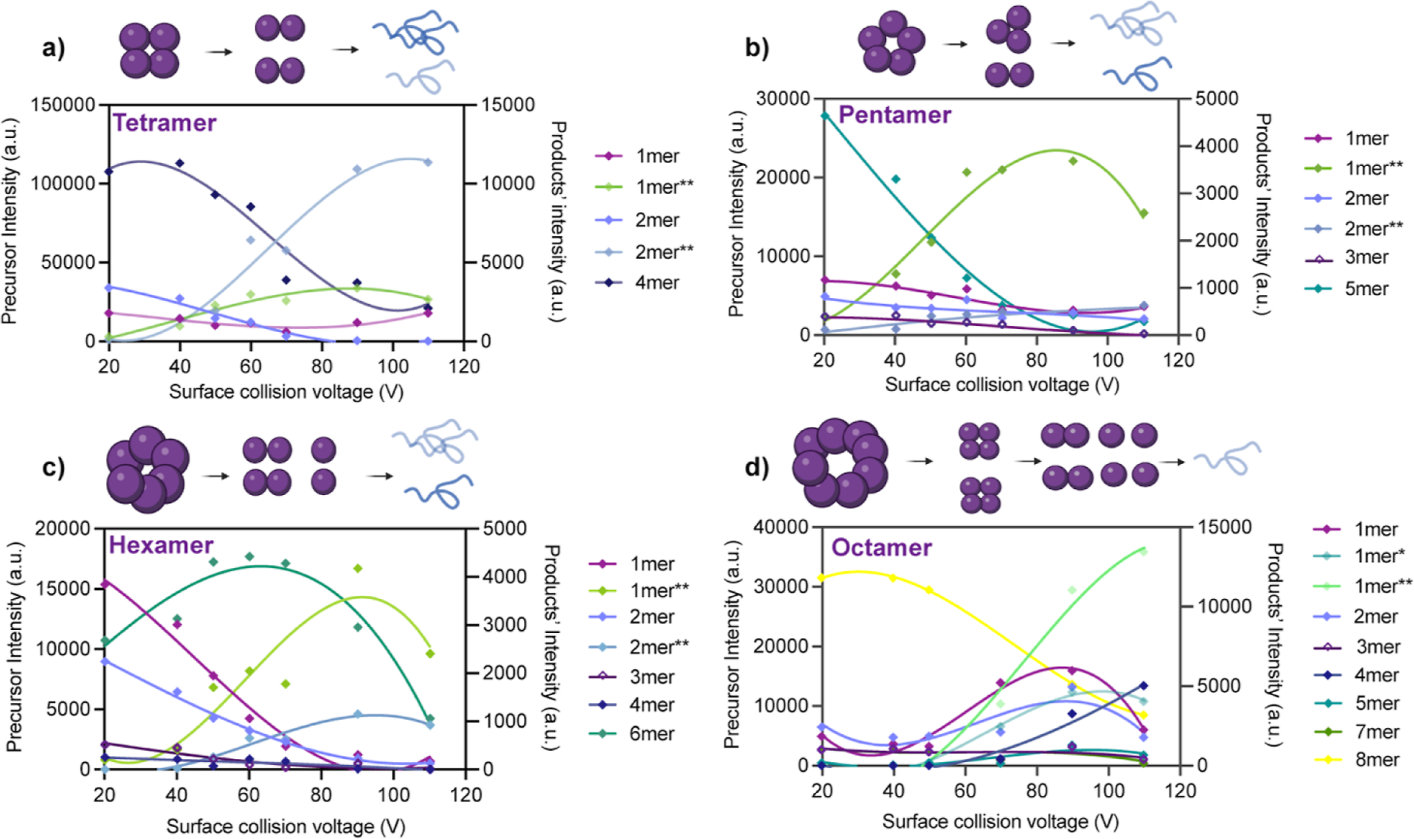
SID profiles of different apo-oligomers. (a–d) Apo-oligomers investigated range from tetramers to octamers. The intensities of all ejected complexes (extracted from their respective IM-MS heat maps) throughout the SID-MS/MS experiment were plotted over collision surface voltage (20 to 110V). Raw intensities of precursor and product ions are traced on the left and right *y*-axis, respectively. The profiles of each population are color-coded to match Figure 4f. Schematics illustrating the most probable dissociation pathway each apo-oligomer are shown above the corresponding plots. In these schemes, the color intensity of each subcomplex reflects its relative abundance, while noncircular shapes represent unfolded species.

Interestingly, most oligomeric complexes preferentially ejected monomers at high SID energies (Figure 5), except for the tetramer, whose main dissociation products were dimers, arising from 40V and withstanding monomer formation at higher collision voltage (Figure 5a). For the 18+ pentamers, at 20V, monomers are the most abundant product while trimers and dimers are also observed (Figure 5b). However, the trimer undergoes rapid dissociation. Since 18+ is one of the lowest charge states of the pentamer, it is most likely to consist of trimers and dimers that subsequently yield unfolded monomers. For the 21+ hexamer, the results show the possibility for multiple subcomplex combinations, namely tetramer and dimer, or two trimers, but mainly a combination of dimers and monomers, as trimers and tetramers are only observed with a low abundance at 20V (Figure 5c). As collisional energy increases, the pattern of unfolded monomer and to a lesser extent dimer ejection dominates. Finally, the 24+ octamer appears to yield multiple dissociation mechanisms (Figure 5d). The most stable octamers are comprised of tetramers that increase and retain their structure until 110V. Secondarily, heptamers and monomers as well as pentamers are visible in lower abundance with minimal increase at higher energies. Dimers and monomers also increase until 90V, where they give rise to unfolded monomeric species.

Overall, for most of the investigated oligomers SID experiments revealed multiple dissociation pathways, suggesting the coexistence of different architectures and highlighting the versatility of those dynamic structures. Interestingly, the dominant apo-oligomers (hexamers and octamers) display high gas-phase stability. At the subunit level, although unfolding of ejected monomers and dimers can be observed over similar energy ranges (Figure 5a), the intersection of folded and unfolded abundance curves occurs consistently at higher energies for dimers, indicating increased resistance to activation-induced unfolding (Figure 5b,c). Together, these observations suggest the presence of additional stabilizing interactions at the dimer level. To explore the possibility of disulfide linkage-mediated oligomerization we reproduced the temperature ramp for a reduced and alkylated apo-SOD1 sample containing iodoacetamide (IA), thus blocking one of the monomer’s accessible cysteines, otherwise forming an intramolecular disulfide bond. The results showed that the carbamidomethylated monomer did not act as an aggregation intermediate, whereas the free apo-SOD1 did (Figures S17–S20). Nonetheless, stable higher-order oligomers were still sufficiently formed confirming the possibility of disulfide linkages as one of the mechanisms involved in bovine SOD1 higher-order assembly.

### Detecting Labile Regions in Apo-SOD1

As SID experiments revealed the existence of multiple assembly mechanisms, primarily containing trimeric, monomeric and dimeric building blocks, the next step would be to elucidate the regions that could serve as binding interfaces for the assembling subcomplexes. Thus, online limited proteolysis by thermolysin, a thermostable metalloprotease from *Bacillus thermoproteolyticus*, was employed after reaching apo-oligomer formation. Thermolysin hydrolyzes peptide bonds at the N-terminal side of hydrophobic residues (e.g., Leu, Ile, Val, Phe). Oligomeric complexes in solution are commonly stabilized by a network of hydrophobic interactions. Hence, in this experiment proteolysis of labile and exposed hydrophobic regions of apo-monomers can provide valuable insights into identifying possible binding interfaces in solution.

Thermolysin was added to a solution containing apo-SOD1 at an enzyme-to-substrate ratio of 1:10 and heated from 55 to 65 °C at a rate of 0.3 °C min^–1^, to induce the production of mainly apo-monomers. After identification of cleaved segments (Table S7), more enzyme accessible regions seem to be localized on loops V, VI and loop VII, otherwise known as the electrostatic loop, containing a high number of hydrophobic residues possibly participating in interactions inducing oligomer formation.

Complementary insights from thermally induced fragmentation highlight the C-terminal region (residues 140- 151) (Figure 6 and Table S8). Notably, our observations align with single-molecule force spectroscopy data identifying human-SOD1 strands β1-β3 as a stable core, withstanding unfolding, whereas β5-β8 and loop VII show greater conformational plasticity.^73^ Overall, for bovine apo-SOD1 our proteolysis data suggests that loops V and VI, stabilizing the β5-β6 hairpin, and the electrostatic loop are more exposed regions in solution. This destabilization can, in turn, promote multimer formation and potentially prion-like aggregation through conformational templating.^74,75^

**Figure 6:**
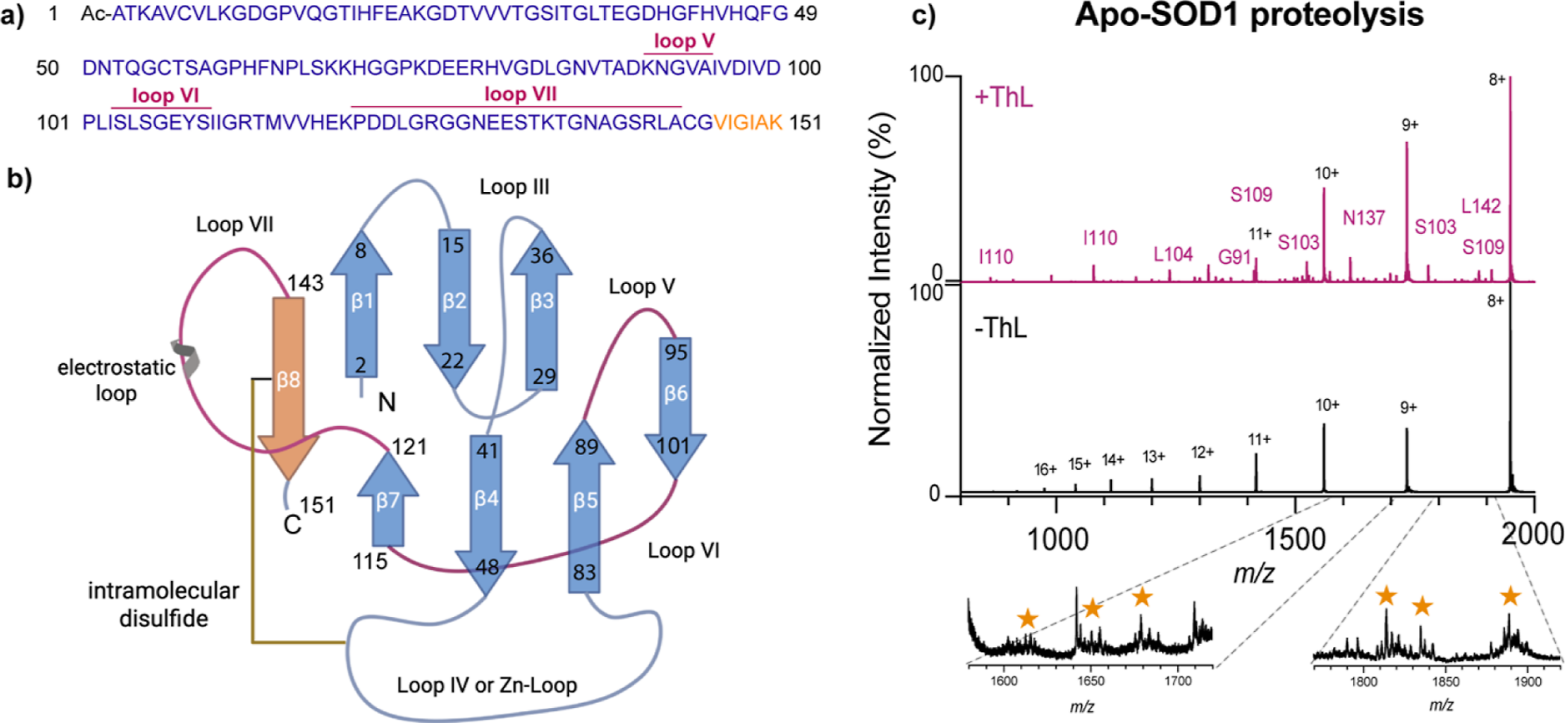
Labile regions of apo-SOD1 revealed via limited proteolysis by thermolysin and heat-induced fragmentation. (a) Sequence of bovine SOD1. Annotated loops indicate regions of cleavage sites after addition of thermolysin, while orange regions were identified as heat-induced fragments. (b) Scheme of the bovine SOD1 structure. The different regions are color-coded, to map loop regions of proteolytic cleavage and heat-induced fragmentation. (c) Spectrum with identified proteolytic fragments of apo-SOD1 monomers after thermolysin addition at 65 °C (top, pink) and spectrum of apo-SOD1 at 65 °C without thermolysin, as a control (bottom, blank). Peaks corresponding to identified cleavage sites are labeled with their positions in the protein sequence. Zoomed view of spectra annotated with orange stars shows low-abundance heat-induced fragments.

## Conclusions

Ion mobility-mass spectrometry is a powerful technology that can unambiguously characterize conformationally diverse copopulated oligomeric protein complexes. This offers unique insights into the events preceding and occurring during early protein aggregation that are thought to be the most important regarding neurodegeneration. However, inducing aggregation often involves long incubation times and a combination of methods to structurally and temporally characterize an ensemble of low-abundance and transient early aggregate formations. In this work, TC-nESI was combined with IM-MS to structurally and kinetically characterize intermediates and oligomeric complexes of bovine SOD1, produced upon heat-accelerated aggregation.

For holo-SOD1, dimer dissociation to monomers of variable folding states initiated oligomerization with fast incorporation of mainly dimers, tetramers and monomers into higher-order assemblies. Interestingly, slower thermal ramping produced more misfolded monomeric SOD1 and enabled more abundant oligomer formation. Apo-SOD1 yielded more abundant and more compact oligomers, and their assembly was more gradual compared to the holo-analogue. Finally, SID-MS/MS experiments revealed multiple dissociation pathways and tetrameric, trimeric, dimeric and monomeric building blocks comprising the scaffold of apo-oligomers, while insights from limited proteolysis and heat-induced fragmentation identified loops III, V, VI and VII, and the C-terminus, as labile regions in misfolded apo-SOD1 monomers. These observations reveal a mechanism involving early native dimer dissociation, monomer misfolding and reassociation to higher-order globular oligomers via hydrophobic interactions localized at defined regions. Additional intermolecular interactions, such as disulfide bridges, between dimers are also suspected to stabilize multimers.

Overall, this study provides the first real-time, high-resolution characterization of early SOD1 misfolding and oligomerization intermediates under controlled thermal stress. By combining TC-nESI with high-resolution IM-MS, SID, and limited proteolysis, we uncover conformationally distinct monomeric and previously unobserved oligomeric species, map labile structural regions driving aggregation, and reveal how metal cofactors modulate complex assembly. The methodology developed in this work establishes an experimental framework to elucidate the structural dynamics of other amyloidogenic proteins, such as mutated human SOD1, amyloid-beta or a-synuclein. Their aggregation pathways often involve metalation-dependent assembly and misfolded monomeric intermediates, making them particularly amenable to this approach.^26,27,76,77^ This strategy helps to bridge a critical gap in understanding the earliest stages of neurodegenerative protein aggregation.

## Supplementary Material



## Data Availability

The original data used in this publication are available in a curated data archive at ETH Zurich (https://www.researchcollection.ethz.ch) under the DOI: 10.3929/ethz-b-000743117.

## Notes

Instrumental parameters: Native IM-MS (Tables S1–S3) and CIU-MS/MS parameters (Table S4); IM-MS data: CCS calculations and distributions for holo-SOD1 (Table S5, Figures S1–S4 & S10–S11); thermal denaturation control and replicate experiments (Figures S5–S8); CIU-MS/MS experiment of apo-monomers (Figure S9); IM-MS data: CCS calculations and distributions for apo-SOD1 (Table S6 & Figures S12–S15); SID-MS/MS for holo-SOD1 (Figure S16); Additional heating experiments of apo- and reduced, alkylated apo-SOD1 (Figures S17–S20); limited proteolysis and thermally induced fragmentation (Tables S7–S8) (PDF)
