# Supplementary material for: Tracking Protein Misfolding and Oligomerization: A Temperature-Controlled Ion Mobility-Mass Spectrometry Approach
Source: Anal Chem. 2026 May 13;98(20):14683–94. doi: 10.1021/acs.analchem.5c06100 (PMC13217362; doi:10.1021/acs.analchem.5c06100)
Supplement: Supplementary file 1 [file ac5c06100_si_001.pdf]

Supporting information

Tracking protein misfolding and oligomerization: A temperature-controlled ion mobility-mass spectrometry approach

Despoina Svingou[1], Luke McAlary[2], Julian Alexander Harrison[1]* and Renato Zenobi[1]*

[1]Laboratory of Organic Chemistry, Department of Chemistry and Applied Biosciences, ETH Zurich, 8093 Zurich, Switzerland

[2]Molecular Horizons and School of Science, Faculty of Science, Medicine and Health, University of Wollongong, Wollongong, NSW 2522, Australia

*Corresponding authors' emails:
Julian Alexander Harrison: harrison@org.chem.ethz.ch
Renato Zenobi: zenobi@org.chem.ethz.ch

Table of Contents

Supplementary note 1. Instrumental parameters 3

Supplementary note 1.1. Native IM-MS parameters 3

Table S1 3
Table S2 4
Table S3 4

Supplementary note 1.2. CIU-MS/MS parameters 5

Table S4 5

Supplementary note 2. Holo-SOD1 5

Figure S1 6
Figure S2 6
Figure S3 7
Figure S4 8
Figure S5 8
Figure S6 9
Figure S7 10
Figure S8 11
Figure S9 11
Figure S10 12
Figure S11 12
Table S5 13

Supplementary note 3. Apo-SOD1 13

Figure S12 14
Figure S13 14
Figure S14 15
Figure S15 15
Table S6 16

Supplementary note 4. SID-MS/MS experiments for holo-SOD1 16

Figure S16 16

Supplementary note 5. Additional control experiments 17

Figure S17 17
Figure S18 17
Figure S19 18
Figure S20 18

Supplementary note 6. Limited proteolysis and thermally induced fragmentation. 19

Table S7 19
Table S8 19

Supplementary note 1. Instrumental parameters

Supplementary note 1.1. Native IM-MS parameters

Table S1. cIMS optimized set of parameters, used for high-mass protein analysis.

NanoLock Spray	Stepwave	Quad/MS Profile/DRE	Trap	Cyclic IMS	Transfer	RF
Capillary (kV): 0.9-1.3	Body Gradient (V): 20	Quadrupole:<br><br>Ion Energy (V): 1.0<br>Pre-filter (V): 5.0	Trap TW Velocity (m/s): 300	Helium cell<br>Helium Entrance (V): 10.0<br>Helium Cell Bias (V): 20.0<br>Helium Exit DC (V): 10.0	Pre Transfer Guide<br><br>Pre ECD Gradient (V): 4.0<br>Pre ECD Bias (V): 3.0<br>Pre Trans Gradient (V): 2.0<br>Pre Trans Bias (V): 3.0	StepWave RF (V): 250
Cone (V): 30	Head Gradient (V): 10	Collision Energy<br><br>Trap CE (V) : 6.0<br>Transfer CE (V) : 5.0	Trap TW Pulse Height (V): 0.1	Pre Array Store<br><br>Pre IMS Reference (V): 85.0<br>Pre Array Gradient (V): 5.0<br>Pre Array Bias (V): 35.0	Transfer (LINAC)<br><br>Transfer Entrance (V): 2.0<br>Transfer Gradient (V): 4.0<br>Transfer Exit (V): 6.0	Ion Guide RF (V): 500
Source offset (V): 10	Ion Guide 1 Offset (V): 5	Detector Voltage (V): 2061	Trap Entrance (V): -0.1	Cyclic IMS<br><br>Entrance (V): 5.0<br>Array Offset (V): 2.0<br>Racetrack Bias (V): 50.0<br>Repeller (V): 100<br>Exit (V): 2.0		Trap RF (V): 400
Source temp. (C): 28	Ion Guide 2 Offset (V): 0.5	DRE Lens<br>pDRE Attenuate: OFF	Trap Bias (V): 1.0	Array TW Velocity (m/s): 375<br>Array TW Height (V): 6.0		Driftcell RF (V): 300
Cone Gas (L/hour): 0	Diff Ap 2 (V): 0.1		Trap DC (V): -1.0	Post Array Store<br><br>Post Array Gradient (V): 25.0<br>Post Array Bias (V): 5.0<br>Post Array DC Exit (-V): 2.0		Pro/Post Array RF (V): 350
Purge gas (L/hour): 0	IG TW Velocity (m/s): 300		Trap Exit (V): 1.0			Cyclic RF (V): 250
NanoFlow Gas (Bar): 0	IG TW Pulse Height: 4.0		Post Trap Gradient (V): 7.0			Transfer RF (V): 800
Reference Capillary (kV): 0	Ion Guide Gas: ON		Post Trap Bias (V): 35.0			Transfer RF Gain: 5
	Ion Guide Gas (mL/min): 30		Collision Gas 1 (mL/min): 10.0<br>Collision Gas 2 (mL/min): 0.0			Ion Guide RF Ramp Enable OFF

Table S2. ADC and global cIMS settings.

ADC settings	TWave parameters
ADC start delay (ms): 15.00	Cyclic TW velocity (m/s): 375
Pushes Per Bin: 1	Array TW velocity (m/s): 375
Number of Bins 200	TW static height (V): 25.0
	TW ramp start height (V):15.0
	TW ramp end height (V): 35.0
	TW ramping rate (V/ms): 2.5

Table S3. Advanced cIMS sequence optimized settings.

Parameter	Inject	Separate	Eject and Acquire
Time	10.00	5.00	40.00
Time Abs	10.00	15.00	63.0
Pre Array Gradient	75.0	75.0	75.0
Pre Array Bias	70.0	75.0	75.0
Array entrance	10	30	50
Wave height	3	0	20
Array offset	50	50	35
Array Mode	Forward	Sideways	Forward Eject
Array Exit	50	50	7
Post Array Gradient	35.0	35.0	35.0
Post Array Bias	25.0	25.0	25.0

Supplementary note 1.2. CIU-MS/MS parameters

IMS data for different trap collisional energies were extracted and represented as 2D contour plots in Figures 2c and 2f using CIUSuite v2.2. DT data was normalized for each voltage step and detected as a feature spot according to the parameters listed in **Table S4**.

Table S4. CIU-MS/MS experimental parameters and processing parameters on CIUSuite v.2.2.

Quadrupole Parameters	CIU Suite parameters
Mode: MS/MS	Smoothing: 2D Savitzky-Golay<br>Window size:5, Iterations:1
Trap CE: 10-40 V	Minimum Feature length: 4
LM Resolution: 6.0	Feature allowed width (ms): 15
HM Resolution: 12.0	Maximum CV Gap length: 5

Supplementary note 2. Holo-SOD1

CCS calibration for holo- and apo-SOD1 employed standards of comparable mass, shape, and charge-state distribution. These standards were measured using the same IM-MS method (Supplementary Note 1.) at waveheights of 24, 25, and 26 V to evaluate reproducibility and determine CCS uncertainty. The curves shown below were generated at 25 V, which was also used for all SOD1 analyses.

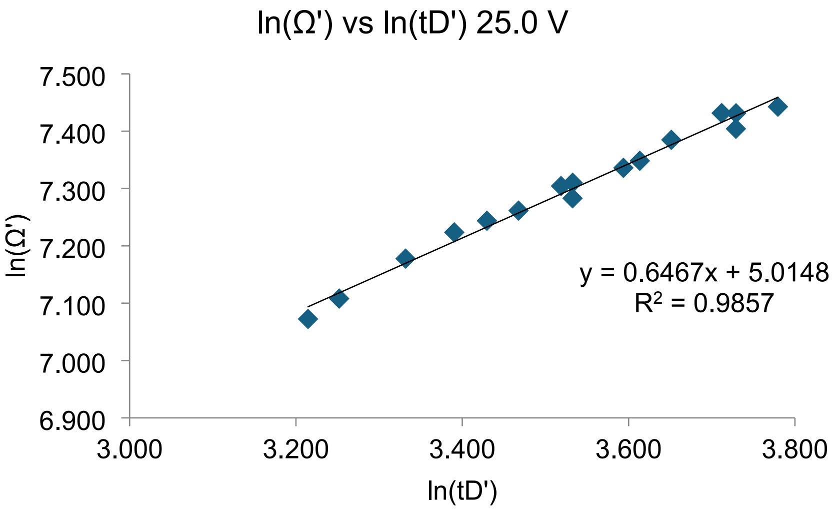

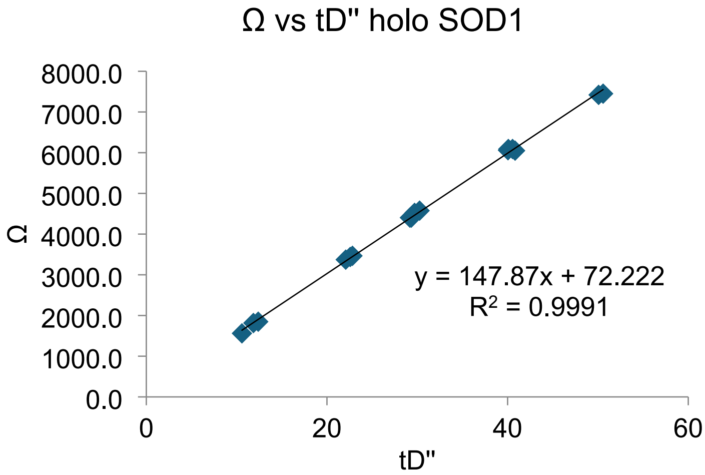

Figure S1. Calibration curves for the CCS calculations of holo-SOD1.

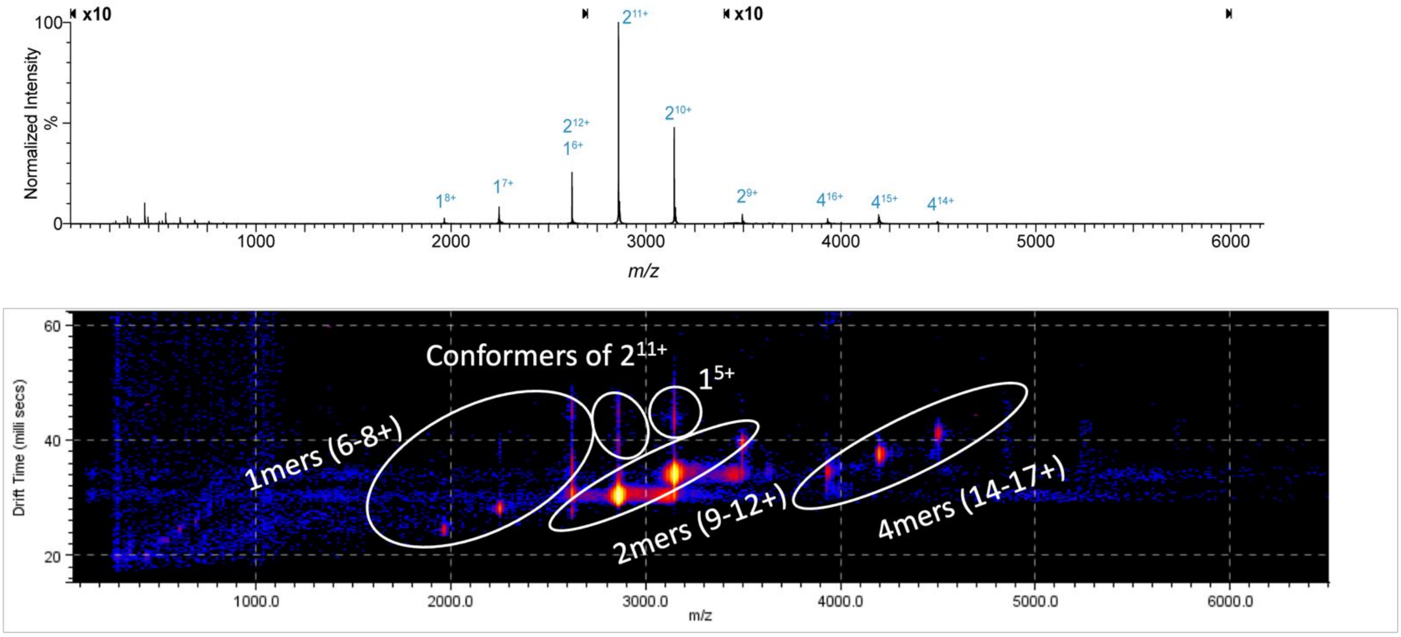

Figure S2. Native spectrum of holo-SOD1 and the corresponding IM-MS heatmap showing initial monomeric, dimeric and tetrameric species and their drift times at the beginning of the fast-heating experiment (at 25 °C). The regions of 50-2700 and 3400-6000 m/z of the mass spectrum are x10 magnified.

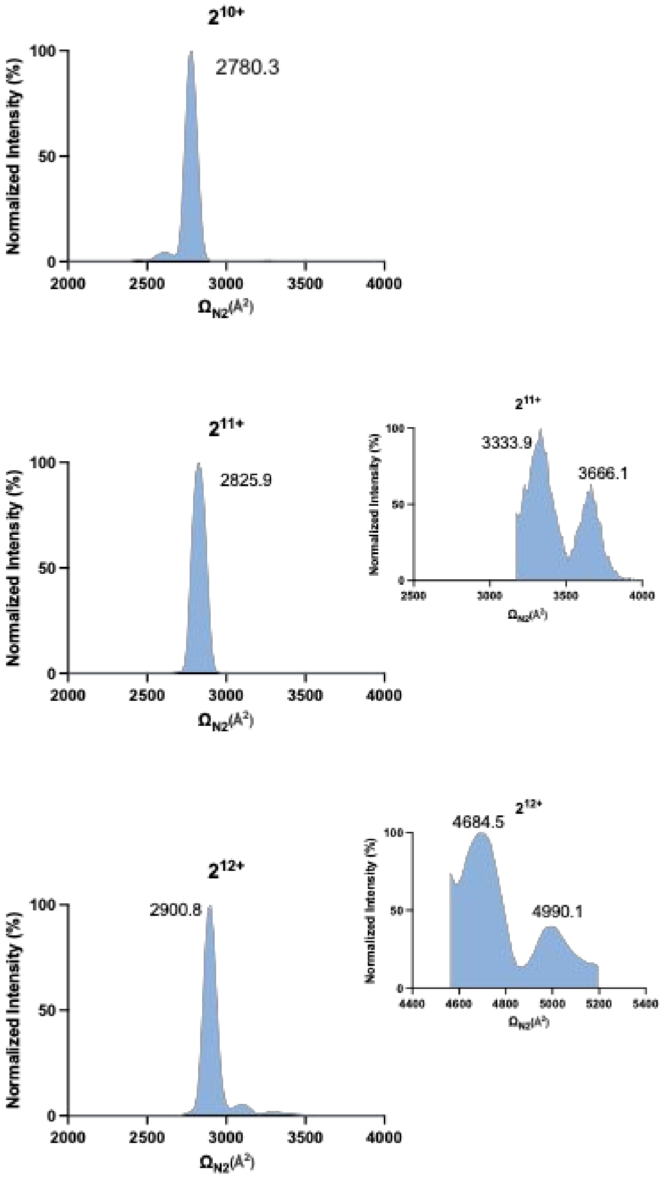

Figure S3. CCS distributions of each charge state of dimeric holo-SOD1, following fast heating (1.0 ºC/min). The presented data were extracted from the corresponding IMS heatmaps, summed over the entire temperature ramp (25-85 °C). The plots in the insets for charge states 11+ and 12+ are zoomed in higher regions of the CCS distributions that reveal minor conformers, resulting from single-pass ion mobility experiments.

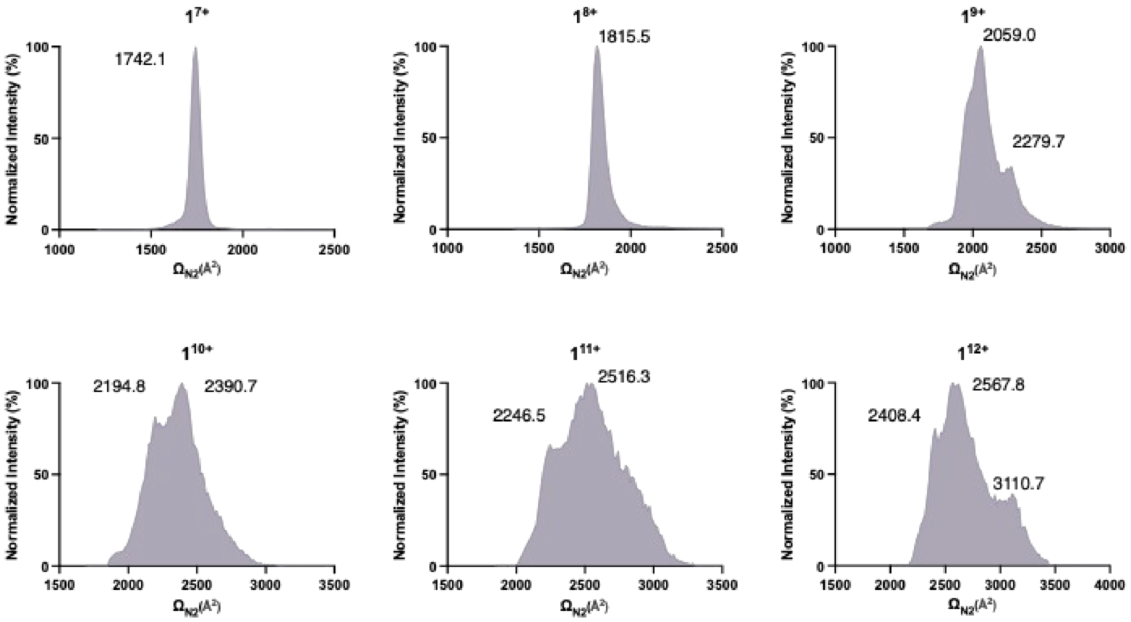

Figure S4. CCS distributions of each charge state of monomeric holo-SOD1, following fast heating (1.0 ºC/min). The presented data were extracted from the corresponding IMS heatmaps, summed over the entire temperature ramp (25-85 °C). The CCS labels correspond to different peaks determined after Gaussian fitting.

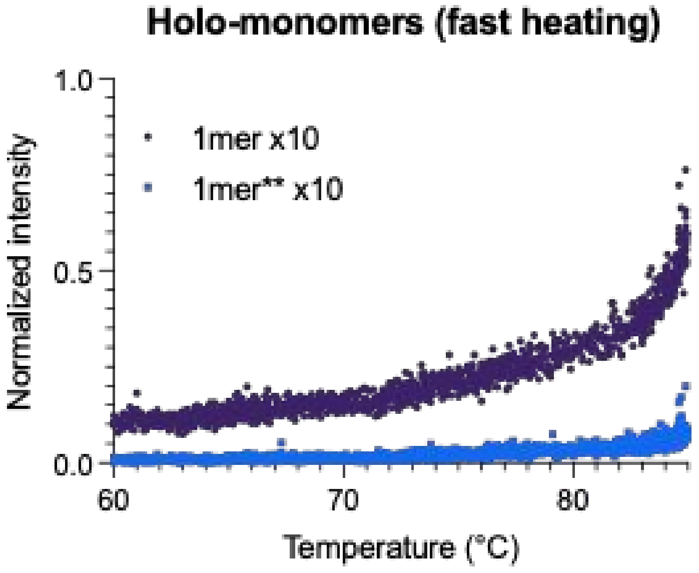

Figure S5. Relative abundance curves of detected monomeric species during the fast heating experiment (25-85 °C, 1 °C/min). Both folded (1mer) and unfolded (1mer**) holo monomers increase simultaneously at high temperature. Both signals are normalized to the TIC and are magnified x10.

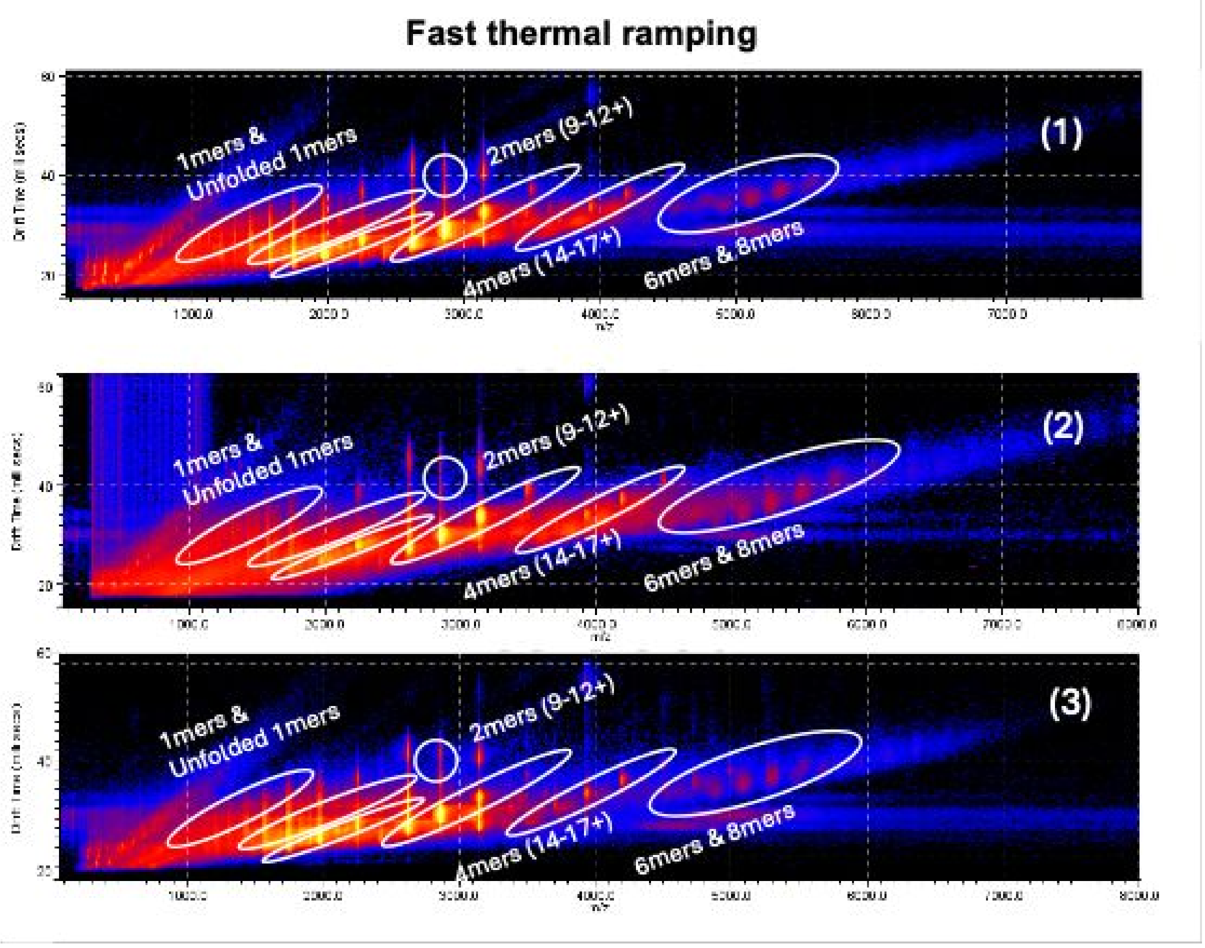

Figure S6. IM-MS heatmaps of summed data from three replicates of fast thermal ramping experiments of holo-SOD1 (35-85 °C, 1.0 °C/min).

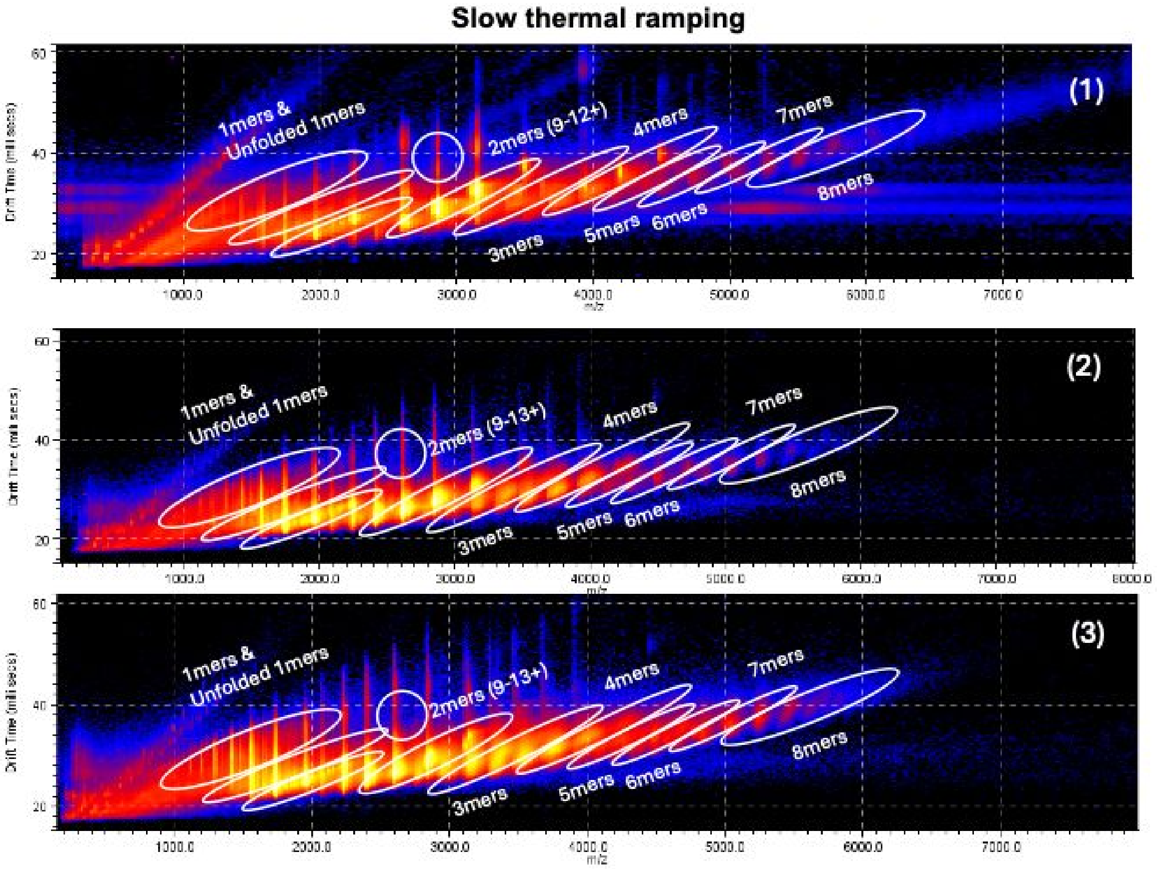

Figure S7. IM-MS heatmaps of summed data from three replicates of slow thermal ramping experiments of holo-SOD1 (35-85 °C, 0.3 °C/min).

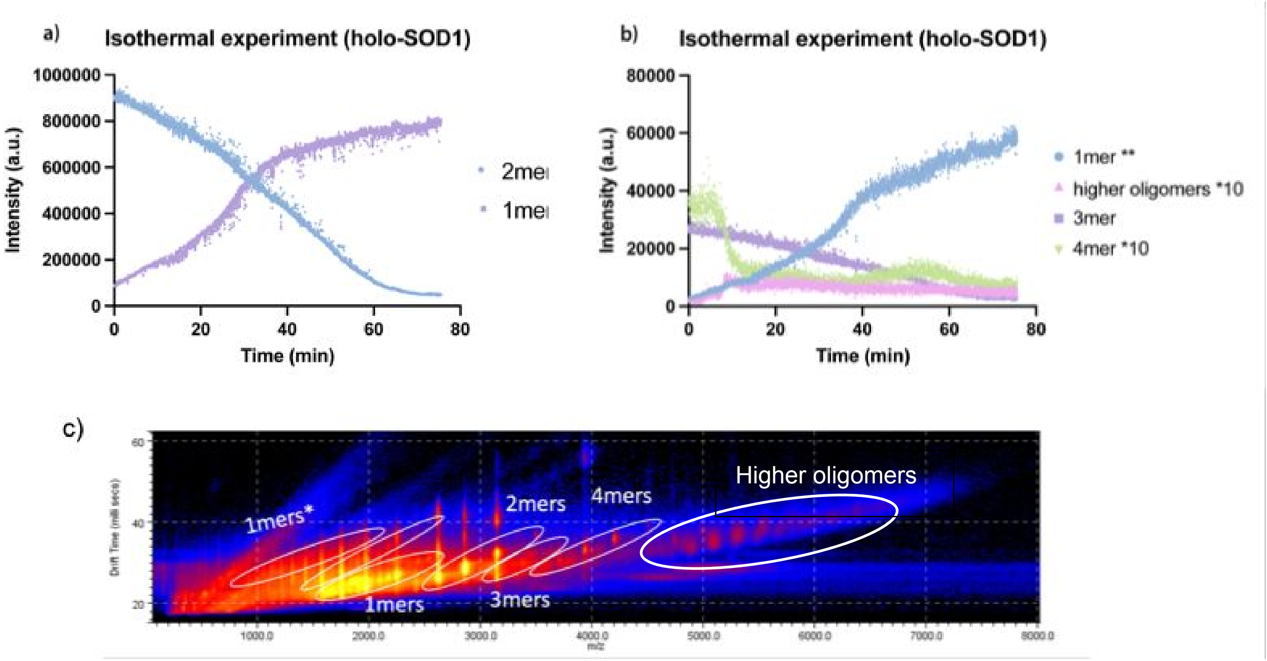

Figure S8. a & b) Abundance profiles of all species over time during the isothermal experiment of holo-SOD1 at 82 °C. c) IM-MS heat map of summed data during the whole isothermal experiment.

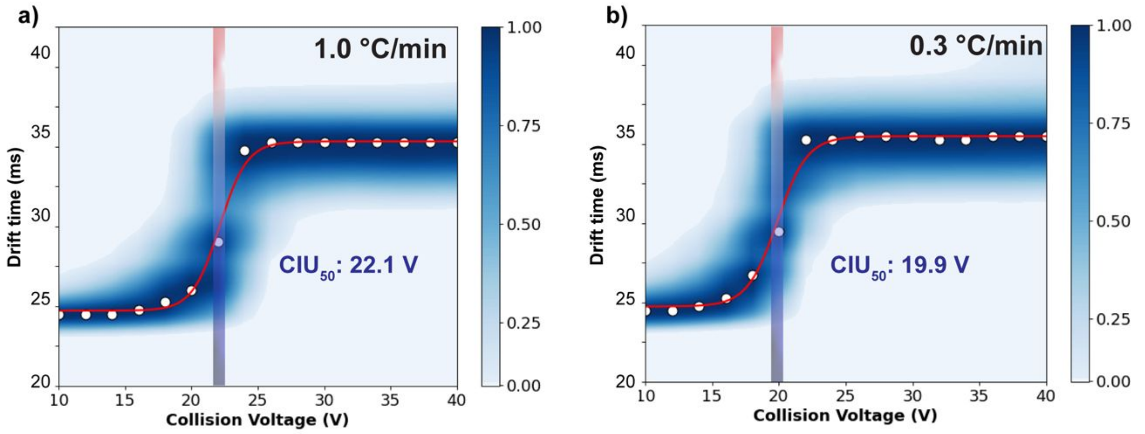

Figure S9. CIU-MS/MS experiment of the 8+ apo-SOD1 monomer after fast (a) and slow (b) thermal ramping in the range of 55-60 °C, 0.3 °C/min.

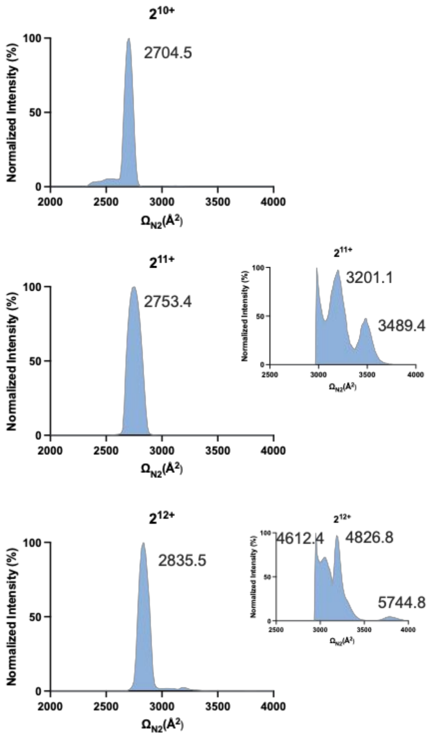

Figure S10. CCS distributions of each charge state of dimeric holo-SOD1, following slow heating (0.3 ºC/min). The presented data were extracted from the corresponding IMS heatmaps, summed over the entire temperature ramp (35-85 °C). The plots in the insets for charge states 11+ and 12+ are zoomed in higher regions of the CCS distributions that reveal minor conformers, resulting from single-pass ion mobility experiments.

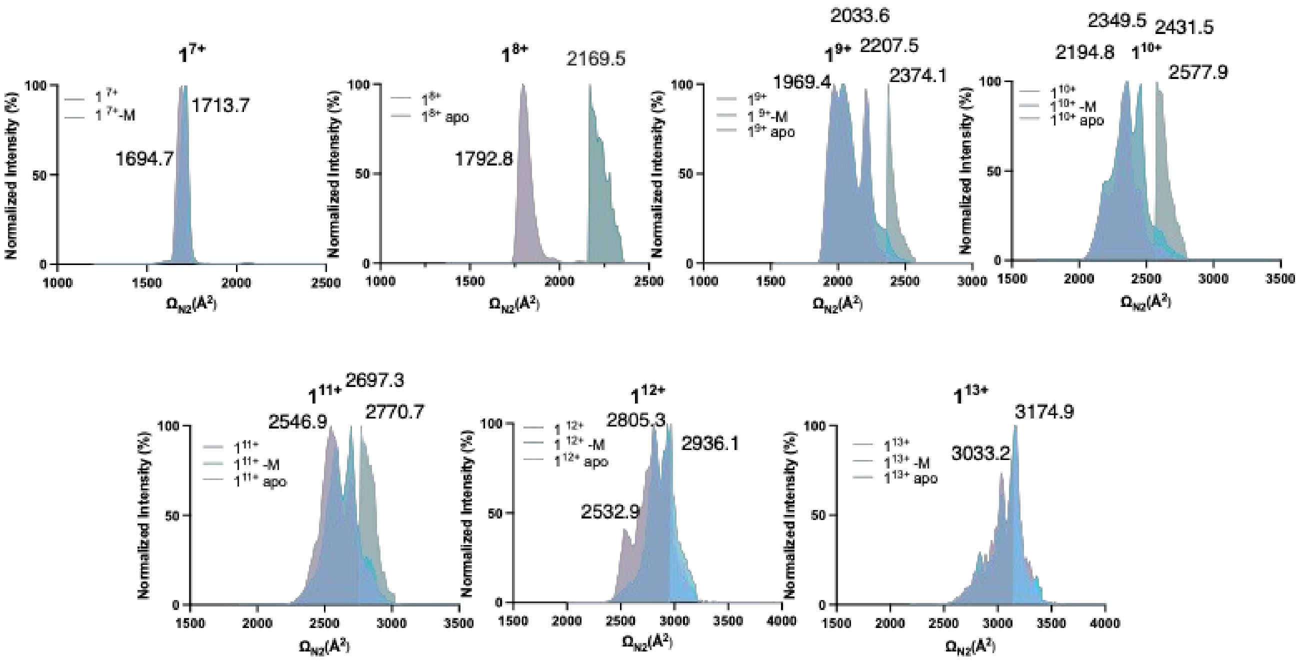

Figure S11. CCS distributions of each charge state of monomeric holo-SOD1, following slow heating (0.3 ºC/min). The presented data were extracted from the corresponding IMS heatmaps, summed over the entire temperature ramp (35-85 °C).

Table S5. CCS values of each charge state of observed holo-SOD1 oligomers, following slow heating (0.3 ºC/min).

Oligomer	Mass (Da)	Charge state	CCS_{N2}(Å^2)
3mer	47147.1	12+	3447.6 ±11.7
		13+	3542.3 ±12.0
		14+	3602.2 ±12.2
4mer	62862.8	14+	4253.2 ±4.7
		15+	4294.1 ±4.7
		16+	4342.5 ±4.8
		17+	4389.8 ±4.8
5mer	78578.5	17+	4514.9 ±11.7
		18+	4906.3 ±12.8
		19+	5406.3 ±18.9
6mer	94294.1	18+	5538.7 ±19.4
		19+	5548.2 ±19.4
		20+	5589.2 ±19.6
		21+	5713.9 ±20.0
8mer	125725.5	22+	6864.1 ±3.4
		23+	6938.9 ±3.5
		24+	6988.6 ±3.5

Supplementary note 3. Apo-SOD1

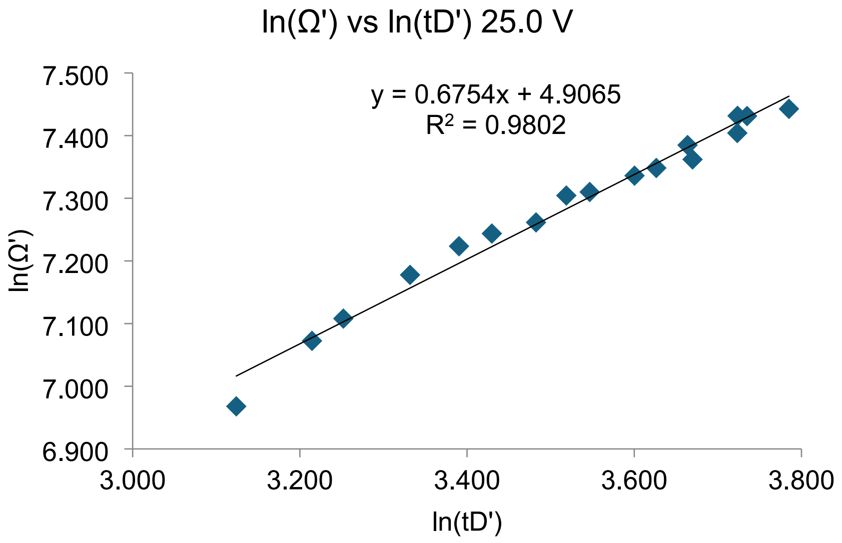

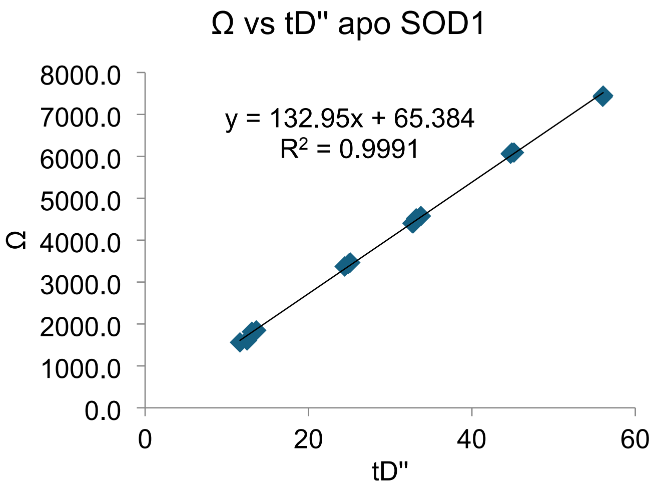

Figure S12. Calibration curves for the CCS calculations of apo-SOD1.

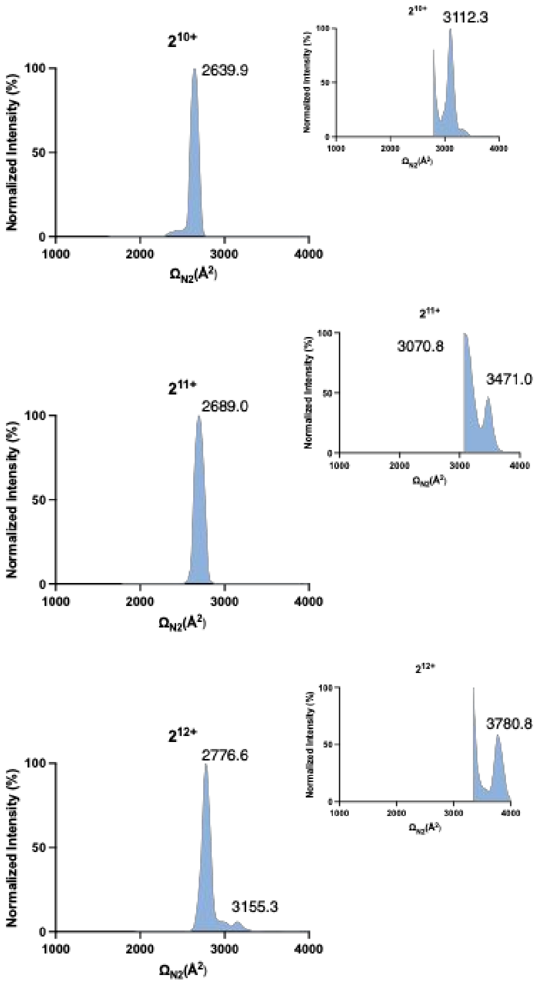

Figure S13. CCS distributions of each charge state of dimeric apo-SOD1, following slow heating (0.3 ºC/min). The presented data were extracted from the corresponding IMS heatmaps, summed over the entire temperature ramp (30-70 °C).

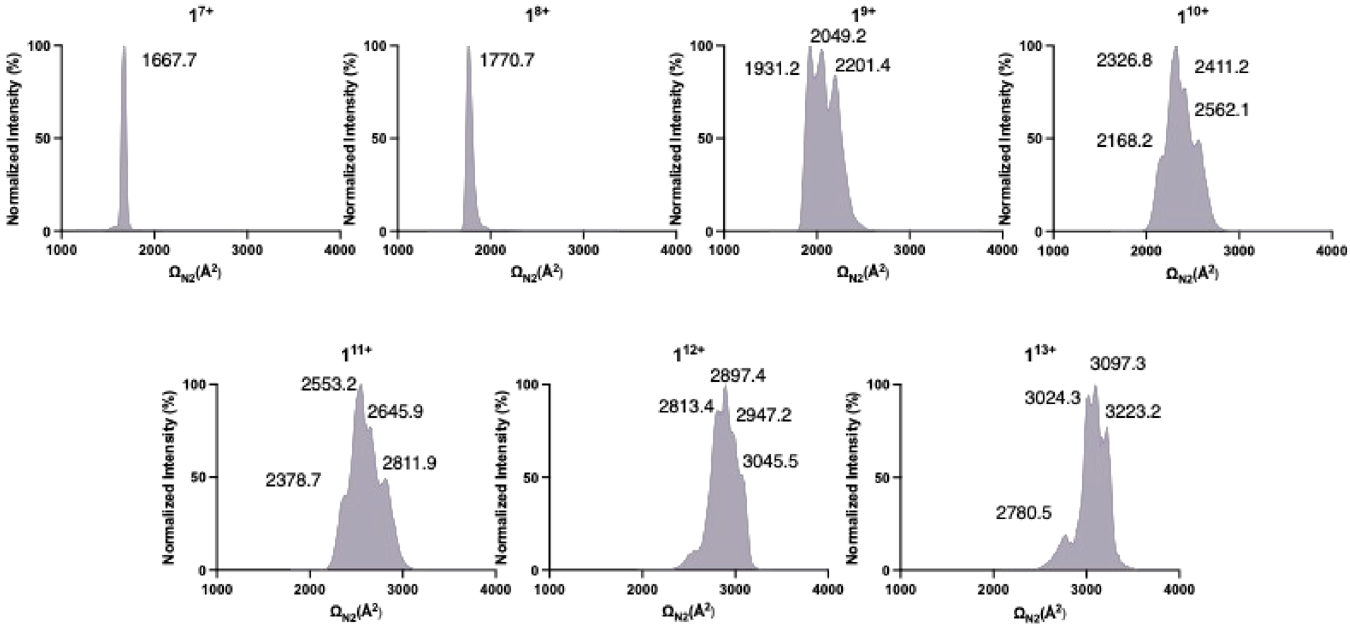

Figure S14. CCS distributions of each charge state of monomeric apo-SOD1, following slow heating (0.3 ºC/min). The presented data were extracted from the corresponding IMS heatmaps, summed over the entire temperature ramp (30-70 °C).

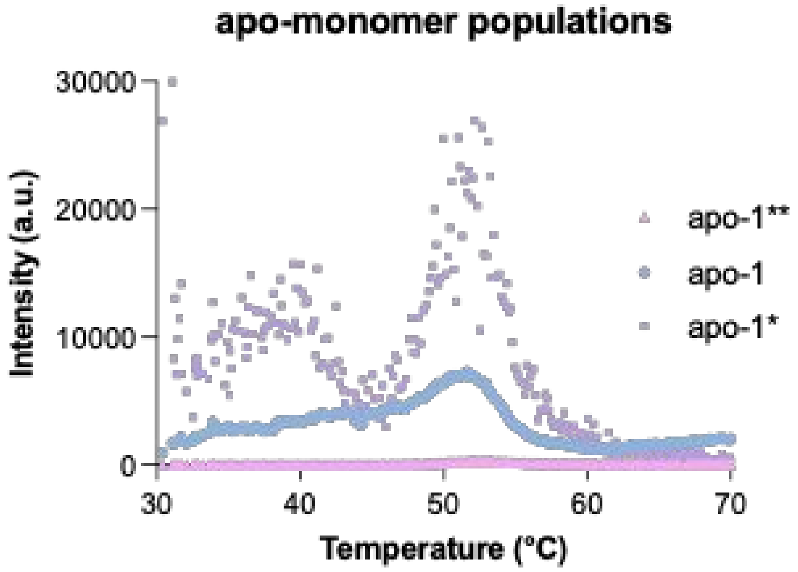

Figure S15. Abundance over temperature profile of different apo-monomeric species during slow heating (0.3 ºC/min).

Table S6. CCS values of each charge state of observed apo-SOD1 oligomers, following slow heating (0.3 ºC/min).

Oligomer	Mass (Da)	Charge state	CCS_{N2}(Å^2)
3mer	46778.6	13+	3359.3 ± 2.4
		14+	3461.2 ± 9.0
		15+	3600.6 ± 9.4
4mer	62371.4	15+	4180.8 ± 3.3
		16+	4161.6 ± 3.3
		17+	4188.1 ± 3.4
5mer	77964.3	17+	4706.8 ± 3.8
		18+	4768.4 ± 3.8
		19+	4827.2 ± 3.9
		20+	5024.1 ± 4.0
6mer	93557.2	19+	5423.1 ± 4.3
		20+	5421.7 ± 4.3
		21+	5496.2 ± 4.4
		22+	5637.9 ± 4.5
7mer	109150	21+	6066.5 ± 18.2
		22+	6071.2 ± 18.2
		23+	6134.5 ± 18.4
		24+	6208.1 ± 18.6
8mer	124743	22+	6462.5 ± 19.4
		23+	6666.7 ± 20.0
		24+	6740.3 ± 15.5
		25+	6825.1 ± 15.7

Supplementary note 4. SID-MS/MS experiments for holo-SOD1.

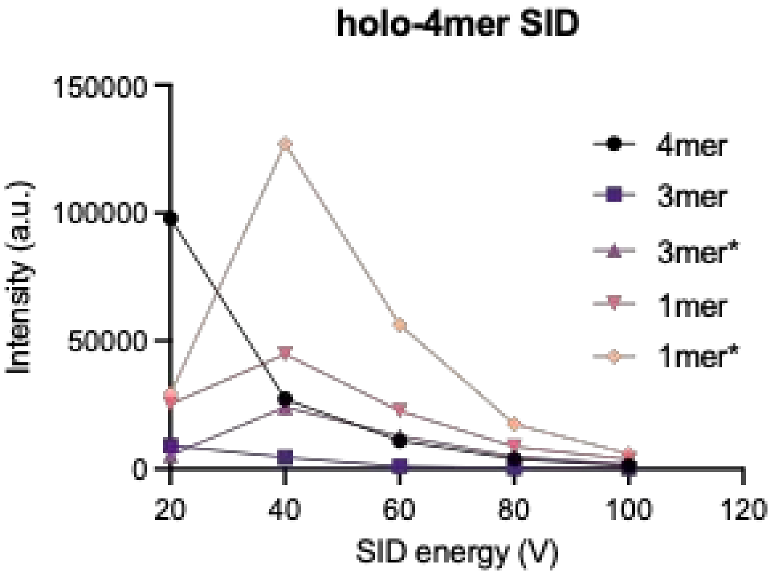

Figure S16. SID-MS/MS results for the 17+ holo-tetramer of holo-SOD1.

Supplementary note 5. Additional control experiments

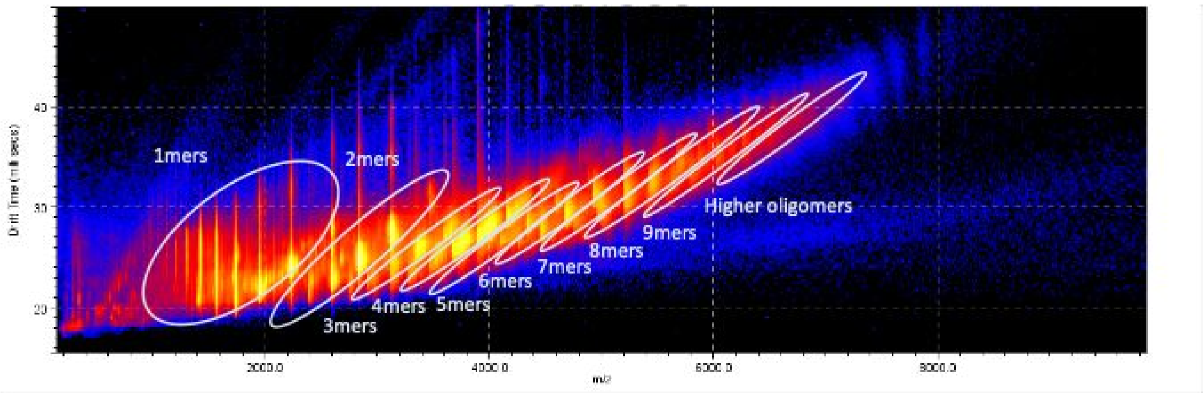

Figure S17. IMS Heat map of a targeted heating thermal denaturation experiment (58-68 ºC) of apo-SOD1 with a slow heating rate (0.3 ºC/min).

Oligomer production is sufficiently increased even though dimer dissociation is accelerated by direct exposure to 58 ºC while slowing down until 68 ºC allows more oligomer formation, avoiding complete monomer unfolding. This shortens the experiment time to 30 min, as opposed to the 2.8-hour long slow heating experiment over 35-85 ºC.

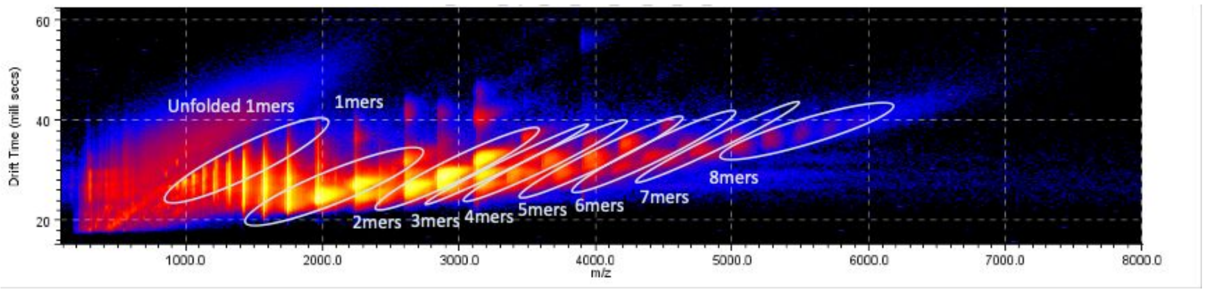

Figure S18. IMS Heat map of a targeted heating thermal denaturation experiment (55-65 ºC) of reduced and alkylated apo-SOD1 with a slow heating rate (0.3 ºC/min).

Reduction was achieved by incubation for 30 min in 60 ºC in a 10 mM DTT solution. The sample was subsequently alkylated by addition of 20mM iodoacetamide solution (IA) at room temperature and in the dark, for another 30 minutes. The reaction was quenched by addition of 10 mM DTT solution and the protein was then subjected to slow heating (0.3 ºC/min) from 55 to 65 ºC. Oligomer production was still apparent, even though monomers presented mostly adducts containing one IA molecule bound per monomer, hindering higher-order complexation by covalent disulfide linkages.

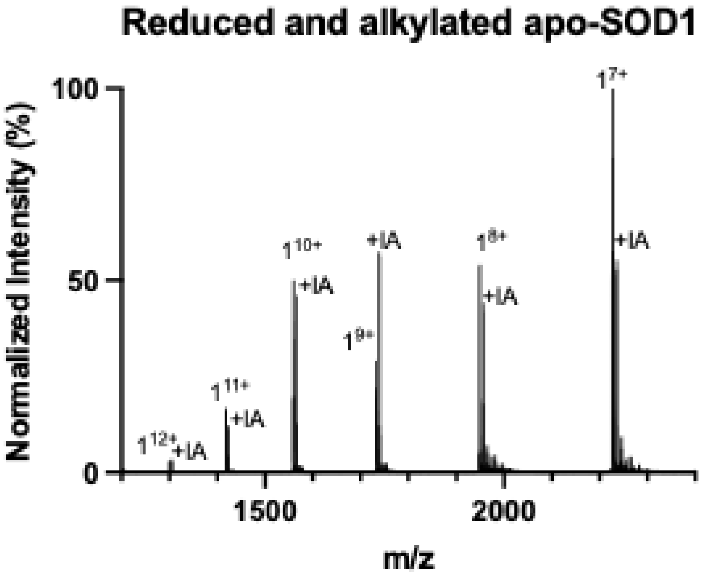

Figure S19. Spectrum of reduced and alkylated apo-SOD1. Monomers and their corresponding IA adducts are annotated.

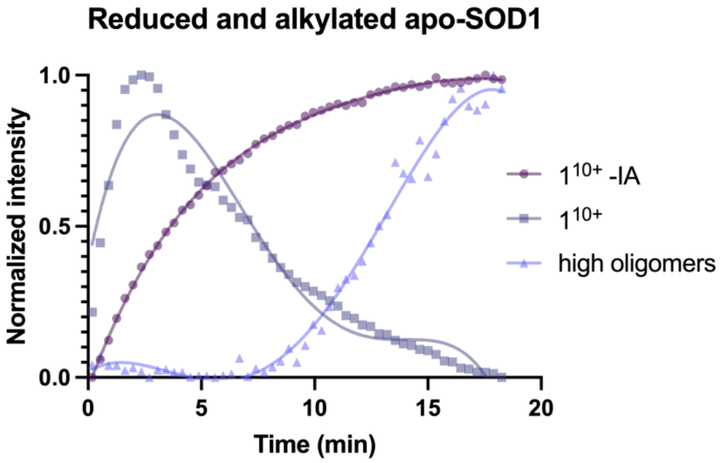

Figure S20. Normalized abundance profiles of reduced and alkylated apo-SOD1 monomer 10+, monomer 10+ bound to IA and higher-order oligomers.

As the plot suggests the 10+ monomer is an intermediate and an initiator for high-order complex formation, whereas alkylated 10+ keeps rising throughout the ramp, hinting at the possibility of disulfide linkage formation of the remaining, non-alkylated apo-SOD1 monomers.

Supplementary note 6. Limited proteolysis and thermally induced fragmentation.

Table S7. Identified cleavage sites of apo-SOD1 by Thermolysin.

Cleavage site	Deconvoluted mass (Da)	Theoretical m/z	Experimental m/z	Charge state	Error (ppm)
A143	830.5	416.2373	416.2350	2+	-5.526
A138	1315	658.3573	658.3510	2+	-9.570
I110	4306	1077.507	1077.517	4+	9.002
		862.207	862.2197	5+	14.38
L104	4944	1237.007	1237.008	4+	0.6467
G91	9424	1347.293	1347.320	7+	19.81
S103	10657	1777.174	1777.147	6+	-15.17
		1523.436	1523.411	7+	-16.39
S109	11294	1883.341	1883.373	6+	17.29
		1412.757	1412.766	8+	6.158
N137	14287	1588.452	1588.451	9+	-0.7205
L142	14772	1847.507	1847.479	8+	-15.32

Table S8. Identified thermally-induced fragments apo-SOD1 (extracted from deconvoluted spectra).

Sequence	Location of cleavage	Experimental deconvoluted mass (Da)	Theoretical Average mass (Da)	Cysteines' status
ATKA…AGSRLACGVIGIAK	C-term	14829.66±51.99	14849.39	Oxidized
ATKA…AGSRLACGVIGIAK	C-term	14513.28±29.14	14510.96	Reduced
ATKA…AGSRLACGVIGIAK	C-term	15103.94±2.00	15106.71	Oxidized
